# Tomosynthesis vs Digital Mammography Screening in Women with a Family History of Breast Cancer

**DOI:** 10.1001/jamaoncol.2025.1209

**Published:** 2025-05-22

**Authors:** Tong Li, Yu-Ru Su, Janie M. Lee, Ellen S. O’Meara, Diana L. Miglioretti, Karla Kerlikowske, Louise Henderson, Nehmat Houssami

**Affiliations:** 1 The Daffodil Centre, The University of Sydney, a Joint Venture with Cancer Council NSW, Sydney, Australia; 2 Kaiser Permanente Washington Health Research Institute, Seattle; 3 Department of Radiology, University of Washington and Fred Hutchinson Cancer Center, Seattle; 4 Division of Biostatistics, Department of Public Health Sciences, University of California, Davis; 5 Department of Epidemiology and Biostatistics, University of California, San Francisco; 6 Department of Medicine, University of California, San Francisco; 7 Department of Radiology, University of North Carolina at Chapel Hill; 8 School of Public Health, Faculty of Medicine and Health, The University of Sydney, Sydney, Australia

## Abstract

**Importance:**

Evidence on screening outcomes with digital breast tomosynthesis (DBT) vs digital mammography (DM) in women with a family history of breast cancer is limited.

**Objective:**

To compare the performance of DBT and DM screening in women with a family history of breast cancer overall and subdivided by breast cancer family history category, breast density, age group, screening interval, and screening round, and to describe characteristics of cancers detected on screening vs interval cancers.

**Design, Setting, and Participants:**

In this comparative cohort study at imaging facilities affiliated with the Breast Cancer Surveillance Consortium, adult women 18 years and older with a self-reported family history of breast cancer who underwent DBT or DM from 2011 to 2018 were included, with a 1-year follow-up for breast carcinoma. Data analysis was performed between November 2023 and August 2024.

**Exposures:**

DBT or DM.

**Main Outcomes and Measures:**

The main outcomes were absolute risk difference (ARD) between DBT and DM for recall rate, cancer detection rate, interval cancer rate, advanced cancer rate, biopsy rate, positive predictive values, sensitivity, and specificity, with inverse probability of treatment weighting.

**Results:**

A total of 208 945 women with a family history of breast cancer undergoing 502 357 screening examinations were included in the sample. Median (IQR) age was 58 (50-66) and 57 (49-66) years for the DBT and DM groups, respectively. Adjusted ARDs (DBT vs DM) were significant for recall rate (−1.51%; 95% CI, −2.42% to −0.59%) and specificity (1.56%; 95% CI, 0.65%-2.46%) in the overall cohort of 121 698 DBT and 380 561 DM examinations and among women with 1 first-degree relative (recall rate ARD, −1.72%; 95% CI, −2.70% to −0.74%; specificity ARD, 1.75%; 95% CI, 0.81%-2.69%). Among those with only second-degree relatives, the biopsy rate for DBT was significantly higher (ARD, 0.39%; 95% CI, 0.18%-0.61%). Significant ARDs were observed for the ductal carcinoma in situ detection rate (−0.71 per 1000 examinations; 95% CI, −1.03 to −0.38 per 1000 examinations) in women with almost entirely fatty breasts; recall rate (−1.90%; 95% CI, −2.88% to −0.92%) and specificity (1.93%; 95% CI, 0.97%-2.89%) in women with scattered fibroglandular densities. Significant ARDs were also observed for the positive predictive value for recall (1.75%; 95% CI, 0.84%-2.67%) in heterogeneously dense breasts, as well as the biopsy rate (0.48%; 95% CI, 0.16%-0.80%) and advanced cancer rate (−0.61 per 1000 examinations; 95% CI, −1.02 to −0.20 per 1000 examinations) in extremely dense breasts. DBT screening had a higher proportion than DM of screen-detected early-stage, invasive cancers with favorable prognostic characteristics.

**Conclusions and Relevance:**

In this cohort study of women with a family history of breast cancer, DBT screening reduced recall rates and increased specificity compared to DM, particularly in women with 1 first-degree relative with breast cancer and those with scattered fibroglandular breast density, and reduced advanced cancer rates in women with extremely dense breasts.

## Key Points

**Question:**

Does performance of digital breast tomosynthesis differ from conventional digital mammography in screening women with a family history of breast cancer?

**Findings:**

This comparative cohort study of 208 945 women with a family history of breast cancer undergoing 502 357 screening examinations with a 1-year cancer follow-up found that digital breast tomosynthesis significantly reduced recall rates and increased specificity.

**Meaning:**

Screening with digital breast tomosynthesis may result in fewer harms than digital mammography for women with a family history of breast cancer.

## Teaser

This cohort study compares the performance of digital breast tomosynthesis to conventional digital mammography in screening women with a family history of breast cancer.

## Introduction

Family history of breast cancer is a well-established risk factor for breast cancer. In the US, approximately 8% to 11% of individuals report having a close family member diagnosed with breast cancer.^1,2^ A meta-analysis of 74 studies confirmed that a family history of breast cancer increases breast cancer risk, with relative risks of 1.5 to 3.6 depending on affected relatives.^3^ A reanalysis of 52 epidemiological studies demonstrated that risk ratios increased with number of affected first-degree relatives, from 1.8 for 1 first-degree relative to 2.9 for 2 first-degree relatives, and 3.9 for 3 first-degree relatives.^4^ Additionally, women with a family history of breast cancer are more likely to have high breast density^5,6^ which can mask cancers on mammography.

Digital breast tomosynthesis (DBT) has been increasingly used in breast screening and shown to improve detection and reduce recall compared to conventional digital mammography (DM),^7,8^ particularly in young women.^9,10^ By enhancing visualization of lesions that may be obscured by dense tissue in DM, DBT detects more cancers in both fatty and dense breasts, and also improves cancer detection in women with dense compared with fatty breasts.^11^ It is not known whether DBT’s performance varies by breast cancer family history category and breast density classification among women with a family history of breast cancer. A recent systematic review highlighted the scarcity of evidence on DBT vs DM in women with a family history of breast cancer and emphasized the need for research using large sample sizes in this population group.^12^

The purpose of this study was to (1) compare performance of DBT and DM for screening women with a family history of breast cancer overall and stratified by breast cancer family history category (degree and number of affected relatives), breast density, age group, screening interval, and screening round, and (2) examine characteristics of cancers detected during screening vs interval cancers in these women.

## Methods

### Study Design and Cohort

We conducted a retrospective analysis using an observational cohort collected from 5 breast imaging registries within the Breast Cancer Surveillance Consortium (BCSC).^13^ Each BCSC registry and the statistical coordinating center received institutional review board approval from their respective institutions for all study procedures. All procedures are compliant with the Health Insurance Portability and Accountability Act. All registries and the statistical coordinating center received a Federal Certificate of Confidentiality and other protections for the identities of women, physicians, and facilities in this research including passive permission, a waiver of consent, or a mix of active consent, passive permission, or a waiver of consent depending on the BCSC site and year.

The study cohort included all female individuals 18 years and older (hereafter, referred to as women) with self-reported family history of breast cancer who underwent either DBT or DM screening from 2011 to 2018 (Figure). A family history of breast cancer was defined as a history of breast cancer in at least 1 first-degree (mother, sister, daughter) or second-degree (grandmother, aunt) female relative (eMethods 1 in Supplement 1).

**Figure.**
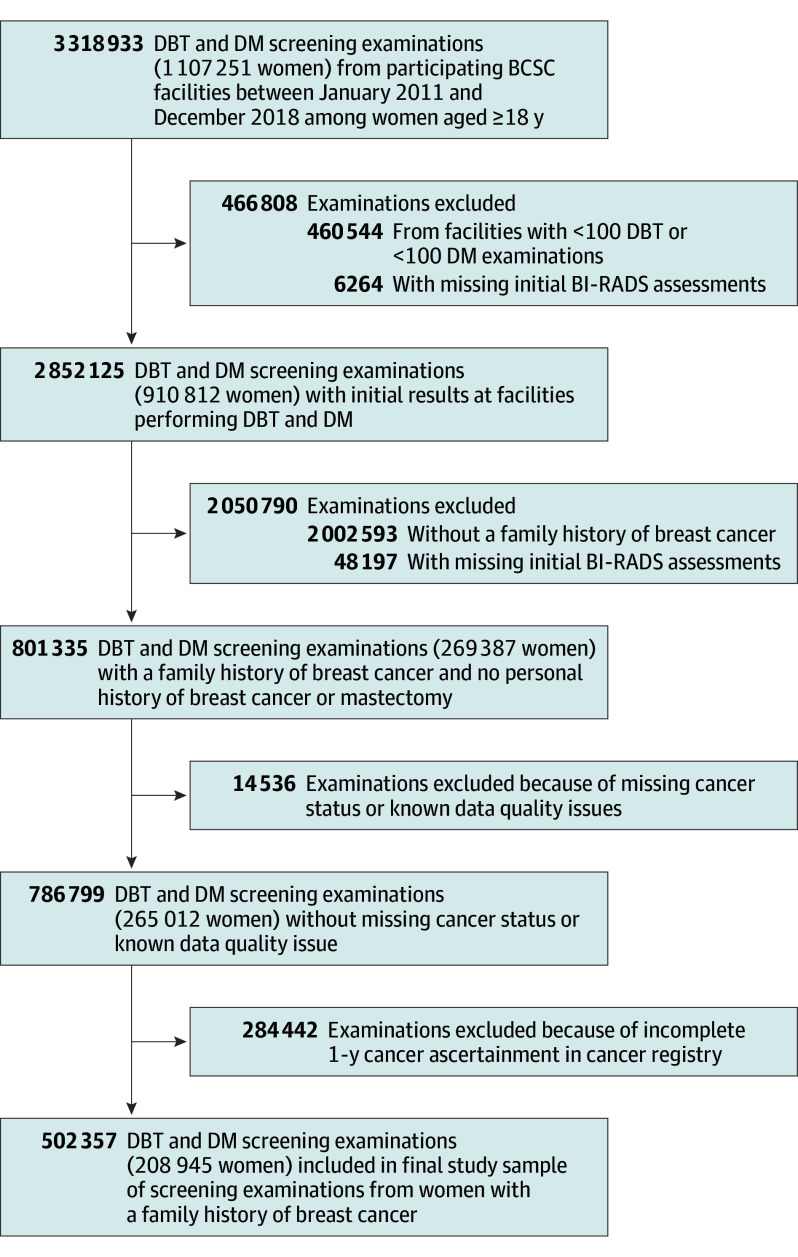
Flow Diagram for Study Population BCSC indicates Breast Cancer Surveillance Consortium; BI-RADS, Breast Imaging–Reporting and Data System; DBT, digital breast tomosynthesis; DM, digital mammography.

Demographic and breast health information was obtained through self-administered questionnaire at the time of screening or extracted from electronic health records. Women were asked if any blood relative had been diagnosed with breast cancer.^14,15^ Self-reported family history of breast cancer was categorized into 3 groups: at least 2 first-degree relatives, 1 first-degree relative, and second-degree relatives only with no known first-degree relatives. Further details on the data collected are in eMethods 2 in Supplement 1.

This study adheres to BCSC definitions^16^ for performance metrics, including recall rate, cancer detection rate (CDR), cancer rate, interval cancer rate (ICR), advanced cancer rate (ACR), biopsy rate, false-positive biopsy recommendation rate, positive predictive values (PPVs), sensitivity, and specificity (eMethods 3 in Supplement 1). Each examination was followed for 12 months or until next screening mammogram, whichever occurred first, for ascertainment of cancer status.^16,17,18^

### Statistical Analysis

Characteristics of screening mammograms were described by imaging modality, and differences between DBT and DM examinations were summarized using standardized mean difference. To adjust for potential confounding from differential characteristics between DBT and DM groups, we applied an inverse probability of treatment weighting (IPTW) approach based on propensity score of receiving DBT at examination level. Propensity score models (PSMs) for DBT vs DM were estimated using logistic regression of modality on age group, breast cancer family history category, race and ethnicity, prior benign breast biopsy result, breast density, screening interval, screening round, and BCSC registry. We followed practical guidelines to select variables for PSMs by including variables related to outcomes or to both outcomes and modality.^19^ An interaction between BCSC registry and breast cancer family history category was included in the PSM for stratified analysis by breast cancer family history category to ensure the balance of BCSC registries between DBT and DM within each breast cancer family history stratum. We evaluated the PSM first by visually comparing weighted density curves of the propensity score in DBT vs DM groups to check for common support,^20^ then evaluated whether the characteristic imbalance was ignorable after IPTW, using a standardized mean difference threshold of less than 25% (eMethods 4 in Supplement 1).^21^

Performance measures were compared between DBT and DM at examination level, and women could have contributed more than 1 examination during the study period. For each measure, the rate in each modality group and absolute risk difference (ARD) between groups were calculated by fitting an IPTW-weighted log-binomial regression with generalized estimation equations with robust sandwich variance estimates to account for clustering by facility. The 95% CI of ARD was derived using the delta method. Analyses on performance measures were conducted in the overall cohort and in subgroups stratified by breast cancer family history category, breast density, age group, screening interval, and screening round. Cancer characteristics were described for DBT and DM groups, and categorized for screen-detected and interval cancers, respectively. To adjust for multiple comparisons, we used the Benjamini-Hochberg procedure^22^ to control false discovery rate at a level of .05 when identifying statistically significant ARDs. Sensitivity analyses are detailed in eMethods 5 in Supplement 1. All statistical analyses were conducted using SAS, version 9.4 (SAS Institute Inc) and R, version 4.0.2 (R Project for Statistical Computing). Data were analyzed between November 2023 and August 2024.

## Results

### Characteristics of Study Cohort

The cohort comprised 502 357 screening examinations (121 790 DBT and 380 567 DM) with 1-year complete cancer follow-up from 208 945 women with a family history of breast cancer (Table 1). Median (IQR) age was 58 (50-66) years and 57 (49-66) years for DBT and DM groups, respectively. Overall, 4.4% of examinations were performed in Asian or Pacific Islander women, 13.3% in Black women, 4.4% in Hispanic or Latina women, 75.1% in White women, and 2.8% in women of unknown or other race, which included Alaska Native, American Indian, multiracial, or self-reported other race.

**Table 1.** Characteristics of Examinations From Women With a Family History of Breast Cancer

Characteristic^a^	Screening modality, No. (%)		Standardized mean difference, %
	DM (n = 380 567)	DBT (n = 121 790)	
No. of women	181 111	68 475	NA
Age group, y			
<40	9693 (2.5)	2873 (2.4)	−1.22
40-49	89 733 (23.6)	27 012 (22.2)	−3.33
50-59	116 521 (30.6)	38 294 (31.4)	1.78
60-69	99 009 (26.0)	34 111 (28.0)	4.49
70-74	31 262 (8.2)	10 395 (8.5)	1.16
≥75	34 349 (9.0)	9105 (7.5)	−5.63
Family history of breast cancer			
≥2 First-degree relatives	11 696 (3.1)	4760 (3.9)	4.55
1 First-degree relative	205 035 (53.9)	65 886 (54.1)	0.45
Second-degree relative(s) only, with no known first-degree relative	163 836 (43.1)	51 144 (42.0)	−2.14
Race and ethnicity			
Asian or Pacific Islander	19 787 (5.2)	2521 (2.1)	−16.78
Black	59 254 (15.6)	7743 (6.4)	−29.81
Hispanic or Latina	18 930 (5.0)	2957 (2.4)	−13.52
White	272 107 (71.5)	105 221 (86.4)	37.16
Other/unknown^b^	10 489 (2.8)	3348 (2.7)	−0.04
Prior benign breast biopsy result			
Yes	33 267 (8.7)	17 142 (14.1)	16.84
No	282 850 (74.3)	84 083 (69.0)	−11.75
Missing	64 450 (16.9)	20 565 (16.9)	−0.13
BI-RADS breast density			
Almost entirely fatty	33 659 (8.8)	12 346 (10.1)	4.41
Scattered fibroglandular density	167 091 (43.9)	57 729 (47.4)	7.02
Heterogeneously dense	145 968 (38.4)	43 072 (35.4)	−6.20
Extremely dense	29682 (7.8)	8258 (6.8)	−3.92
Missing	4167 (1.1)	385 (0.3)	−9.32
Examination year			
2011-2012	142 940 (37.6)	3398 (2.8)	−96.13
2013-2014	154 583 (40.6)	26 430 (21.7)	−41.73
2015-2016	55 085 (14.5)	33 868 (27.8)	33.10
2017-2018	27 959 (7.3)	58 094 (47.7)	101.27
Screening interval (time since last examination)			
First screens (no previous examination)	20 834 (5.5)	4479 (3.7)	−8.61
Annual (9-18 mo)	259 661 (68.2)	90 416 (74.2)	13.30
Biennial (19-30 mo)	48 480 (12.7)	14 283 (11.7)	−3.09
Triennial or longer (>30 mo)	34 040 (8.9)	10 506 (8.6)	−1.12
Missing	17 552 (4.6)	2106 (1.7)	−16.51
Screening round			
First/prevalent	20 834 (5.5)	4479 (3.7)	−8.61
Incident/subsequent	358 843 (94.3)	116 276 (95.5)	5.36
Missing	890 (0.2)	1035 (0.8)	8.40
Academic facility			
No	337 325 (88.6)	81 212 (66.7)	−54.64
Yes	43 242 (11.4)	40 578 (33.3)	54.64

Abbreviations: BI-RADS, Breast Imaging–Reporting and Data System; DBT, digital breast tomosynthesis; DM, digital mammography; NA, not applicable.

^a^ Demographic and breast health information was obtained through a self-administered questionnaire at the time of screening or extracted from electronic health records.

^b^ Other racial and ethnic groups included Alaska Native, American Indian, multiracial, and self-reported other racial group.

Compared to DM, a greater proportion of DBT examinations were performed in White women (86.4% vs 71.5%), those with prior benign breast biopsy results (14.1% vs 8.7%), and at academic facilities (33.3% vs 11.4%). Conversely, compared to DM, a smaller proportion of DBT examinations were performed in Asian or Pacific Islander (2.1% vs 5.2%), Black (6.4% vs 15.6%), Hispanic or Latina women (2.4% vs 5.0%), and women of unknown or other race (2.7% vs 2.8%). Characteristics of these women at their first available and eligible examination are in eTable 1 in Supplement 1.

### Comparison of Performance Measures

IPTW-adjusted recall rate was significantly lower for DBT than DM with an ARD of −1.51% (95% CI, −2.42% to −0.59%) (Table 2). The higher ARD for DBT vs DM specificity was statistically significant at 1.56% (95% CI, 0.65%-2.46%). No statistically significant ARDs were found between DBT and DM in other measures, including cancer detection metrics.

**Table 2.** Adjusted Performance Measures by Modality^a^

Performance measures	Screening modality, rate or proportion (95% CI)		Absolute risk difference (95% CI)
	DM (n = 380 561)	DBT (n = 121 698)	
Recall rate, %	10.10 (9.21 to 11.07)	8.59 (7.78 to 9.49)	−1.51 (−2.42 to −0.59)^b^
Biopsy rate, %	1.41 (1.23 to 1.62)	1.60 (1.39 to 1.85)	0.19 (−0.01 to 0.38)
False-positive biopsy recommendation rate, %	0.95 (0.80 to 1.12)	1.09 (0.92 to 1.29)	0.14 (−0.01 to 0.30)
Cancer detection rate, per 1000 examinations	5.37 (4.67 to 6.18)	5.72 (5.00 to 6.54)	0.34 (−0.52 to 1.21)
Invasive cancer detection rate, per 1000 examinations	4.13 (3.52 to 4.84)	4.46 (3.97 to 5.02)	0.33 (−0.35 to 1.02)
DCIS detection rate, per 1000 examinations	1.25 (1.05 to 1.49)	1.26 (0.92 to 1.72)	0.01 (−0.41 to 0.43)
Cancer rate, per 1000 examinations	6.33 (5.47 to 7.33)	6.73 (6.04 to 7.50)	0.40 (−0.57 to 1.37)
Interval cancer rate, per 1000 examinations	0.96 (0.76 to 1.22)	1.01 (0.80 to 1.29)	0.05 (−0.27 to 0.38)
Invasive interval cancer rate, per 1000 examinations	0.87 (0.68 to 1.12)	0.82 (0.65 to 1.02)	−0.05 (−0.34 to 0.23)
Total advanced cancer rate, per 1000 examinations	0.53 (0.45 to 0.64)	0.55 (0.39 to 0.79)	0.02 (−0.21 to 0.25)
Screen-detected advanced cancer rate, per 1000 examinations	0.37 (0.30 to 0.46)	0.34 (0.22 to 0.53)	−0.03 (−0.20 to 0.15)
Interval advanced cancer rate, per 1000 examinations	0.17 (0.13 to 0.22)	0.21 (0.13 to 0.35)	0.05 (−0.07 to 0.16)
PPV for recall, %	5.41 (4.71 to 6.21)	6.85 (5.94 to 7.89)	1.44 (0.43 to 2.45)^c^
PPV for biopsy recommended, %	28.91 (25.30 to 33.03)	29.17 (25.64 to 33.17)	0.26 (−3.54 to 4.05)
PPV for biopsy performed, %	33.07 (29.00 to 37.72)	32.11 (28.71 to 35.92)	−0.96 (−5.00 to 3.08)
Sensitivity, %	85.10 (83.05 to 87.19)	85.25 (81.14 to 89.57)	0.16 (−4.33 to 4.64)
Specificity, %	90.39 (89.48 to 91.31)	91.94 (91.10 to 92.79)	1.56 (0.65 to 2.46)^b^

Abbreviations: DBT, digital breast tomosynthesis; DCIS, ductal carcinoma in situ; DM, digital mammography; PPV, positive predictive value.

^a^ Models are adjusted for age group, breast cancer family history category, race and ethnicity, history of benign breast biopsy results, breast density, screening interval, screening round, and Breast Cancer Surveillance Consortium participating registry.

^b^ Absolute risk difference is significant with a false discovery rate threshold of .05 after adjusting for multiple comparisons (using the Benjamini-Hochberg procedure).

^c^ Estimates with corresponding 95% CIs of the absolute risk difference not covering 0, but not significant with a false discovery rate threshold of .05 after adjusting for multiple comparisons (using the Benjamini-Hochberg procedure).

No significant ARDs were found in women with at least 2 first-degree relatives in adjusted measures for DBT and DM (Table 3). For women with 1 first-degree relative, recall rate was significantly lower (ARD, −1.72%; 95% CI, −2.70% to −0.74%) and specificity significantly higher (ARD, 1.75%; 95% CI, 0.81%-2.69%) for DBT vs DM. For women with only second-degree relatives, biopsy (ARD, 0.39%; 95% CI, 0.18%-0.61%) and false-positive biopsy recommendation rates (ARD, 0.28%; 95% CI, 0.09%-0.46%) were significantly higher for DBT vs DM. The remaining measures were not significantly different between DBT and DM across categories of family history of breast cancer. Sensitivity analysis, stratified by breast cancer family history category and restricted to subsequent examinations (eTable 2 in Supplement 1), showed findings consistent with those using all screening examinations.

**Table 3.** Adjusted Performance Measures Stratified by Breast Cancer Family History Category^a^

Performance measures	Screening modality, rate or proportion (95% CI)				Absolute risk difference (95% CI)
	DM	DBT			
**≥2 First-degree relatives (n = 16 381)**					
Recall rate, %	9.36 (8.21 to 10.67)		8.22 (6.73 to 10.04)	−1.14 (−2.82 to 0.54)	
Biopsy rate, %	1.31 (1.06 to 1.62)		1.32 (1.07 to 1.63)	0.01 (−0.37 to 0.39)	
False-positive biopsy recommendation rate, %	0.74 (0.56 to 0.99)		0.80 (0.58 to 1.12)	0.06 (−0.27 to 0.38)	
Cancer detection rate, per 1000 examinations	6.79 (5.16 to 8.93)		5.83 (3.54 to 9.59)	−0.96 (−4.00 to 2.09)	
Invasive cancer detection rate, per 1000 examinations	4.94 (3.61 to 6.76)		5.19 (3.18 to 8.47)	0.25 (−2.47 to 2.96)	
DCIS detection rate, per 1000 examinations	1.84 (1.25 to 2.72)		0.64 (0.21 to 1.99)	−1.20 (−2.08 to −0.32)^c^	
Cancer rate, per 1000 examinations	8.17 (6.10 to 10.95)		8.28 (6.21 to 11.05)	0.11 (−2.74 to 2.96)	
Interval cancer rate, per 1000 examinations	1.39 (0.75 to 2.55)		2.45 (1.23 to 4.88)	1.07 (−0.75 to 2.89)	
Invasive interval cancer rate, per 1000 examinations	1.39 (0.75 to 2.55)		0.57 (0.21 to 1.51)	−0.82 (−1.92 to 0.28)	
Total advanced cancer rate, per 1000 examinations	0.40 (0.18 to 0.89)		0.71 (0.28 to 1.80)	0.31 (−0.38 to 1.01)	
Screen-detected advanced cancer rate, per 1000 examinations	0.20 (0.07 to 0.57)		0.48 (0.15 to 1.50)	0.27 (−0.30 to 0.85)	
Interval advanced cancer rate, per 1000 examinations	0.19 (0.05 to 0.76)		0.23 (0.04 to 1.47)	0.04 (−0.50 to 0.57)	
PPV for recall, %	7.36 (5.58 to 9.70)		8.06 (5.29 to 12.29)	0.70 (−2.72 to 4.13)	
PPV for biopsy recommended, %	36.25 (28.64 to 45.87)		37.82 (24.61 to 58.14)	1.58 (−13.42 to 16.57)	
PPV for biopsy performed, %	43.28 (35.28 to 53.10)		39.28 (24.67 to 62.56)	−4.00 (−21.90 to 13.90)	
Sensitivity, %	83.69 (76.74 to 91.28)		70.59 (51.80 to 96.20)	−13.10 (−35.45 to 9.25)	
Specificity, %	91.26 (90.06 to 92.47)		92.38 (90.72 to 94.08)	1.12 (−0.58 to 2.82)	
**1 First-degree relative (n = 270 857)**					
Recall rate, %	9.83 (8.94 to 10.82)		8.11 (7.25 to 9.08)	−1.72 (−2.70 to −0.74)^b^	
Biopsy rate, %	1.50 (1.30 to 1.72)		1.57 (1.32 to 1.87)	0.07 (−0.19 to 0.34)	
False-positive biopsy recommendation rate, %	0.95 (0.79 to 1.13)		1.00 (0.81 to 1.24)	0.06 (−0.14 to 0.25)	
Cancer detection rate, per 1000 examinations	6.26 (5.26 to 7.44)		6.37 (5.41 to 7.51)	0.12 (−1.20 to 1.43)	
Invasive cancer detection rate, per 1000 examinations	4.89 (3.96 to 6.05)		5.05 (4.38 to 5.82)	0.15 (−0.97 to 1.28)	
DCIS detection rate, per 1000 examinations	1.36 (1.12 to 1.65)		1.32 (0.93 to 1.88)	−0.04 (−0.55 to 0.47)	
Cancer rate, per 1000 examinations	7.44 (6.19 to 8.94)		7.46 (6.44 to 8.64)	0.02 (−1.55 to 1.60)	
Interval cancer rate, per 1000 examinations	1.18 (0.88 to 1.59)		1.09 (0.82 to 1.45)	−0.09 (−0.56 to 0.38)	
Invasive interval cancer rate, per 1000 examinations	1.08 (0.79 to 1.48)		0.94 (0.72 to 1.23)	−0.14 (−0.57 to 0.29)	
Total advanced cancer rate, per 1000 examinations	0.63 (0.51 to 0.76)		0.66 (0.44 to 1.00)	0.04 (−0.28 to 0.35)	
Screen-detected advanced cancer rate, per 1000 examinations	0.43 (0.34 to 0.55)		0.43 (0.26 to 0.70)	−0.00 (−0.23 to 0.23)	
Interval advanced cancer rate, per 1000 examinations	0.20 (0.14 to 0.27)		0.23 (0.13 to 0.43)	0.04 (−0.13 to 0.20)	
PPV for recall, %	6.47 (5.44 to 7.70)		8.06 (7.00 to 9.27)	1.58 (0.22 to 2.95)^c^	
PPV for biopsy recommended, %	32.07 (27.35 to 37.61)		32.28 (27.65 to 37.69)	0.21 (−5.49 to 5.91)	
PPV for biopsy performed, %	36.78 (31.49 to 42.96)		36.30 (32.07 to 41.08)	−0.49 (−6.29 to 5.32)	
Sensitivity, %	84.29 (81.69 to 86.98)		85.39 (81.38 to 89.59)	1.09 (−3.62 to 5.81)	
Specificity, %	90.73 (89.81 to 91.67)		92.48 (91.62 to 93.35)	1.75 (0.81 to 2.69)^b^	
**Second-degree relative(s) only, with no known first-degree relative(s) (n = 214 958)**					
Recall rate, %	10.50 (9.56 to 11.53)		9.58 (8.21 to 11.17)	−0.92 (−2.37 to 0.53)	
Biopsy rate, %	1.32 (1.13 to 1.54)		1.71 (1.46 to 1.99)	0.39 (0.18 to 0.61)^b^	
False-positive biopsy recommendation rate, %	0.97 (0.80 to 1.16)		1.24 (1.04 to 1.49)	0.28 (0.09 to 0.46)^b^	
Cancer detection rate, per 1000 examinations	4.13 (3.61 to 4.74)		5.09 (4.44 to 5.83)	0.95 (0.25 to 1.66)^c^	
Invasive cancer detection rate, per 1000 examinations	3.08 (2.71 to 3.49)		3.71 (3.21 to 4.29)	0.63 (0.10 to 1.16)^c^	
DCIS detection rate, per 1000 examinations	1.05 (0.83 to 1.34)		1.38 (0.91 to 2.07)	0.32 (−0.25 to 0.89)	
Cancer rate, per 1000 examinations	4.77 (4.17 to 5.45)		5.83 (5.15 to 6.59)	1.06 (0.33 to 1.78)^c^	
Interval cancer rate, per 1000 examinations	0.64 (0.50 to 0.81)		0.74 (0.52 to 1.05)	0.10 (−0.19 to 0.39)	
Invasive interval cancer rate, per 1000 examinations	0.56 (0.44 to 0.72)		0.70 (0.49 to 1.00)	0.14 (−0.14 to 0.42)	
Total advanced cancer rate, per 1000 examinations	0.42 (0.32 to 0.56)		0.42 (0.23 to 0.76)	−0.01 (−0.28 to 0.26)	
Screen-detected advanced cancer rate, per 1000 examinations	0.30 (0.22 to 0.42)		0.20 (0.11 to 0.37)	−0.10 (−0.26 to 0.06)	
Interval advanced cancer rate, per 1000 examinations	0.12 (0.08 to 0.19)		0.21 (0.07 to 0.61)	0.09 (−0.13 to 0.31)	
PPV for recall, %	3.99 (3.49 to 4.57)		5.38 (4.41 to 6.56)	1.39 (0.38 to 2.39)^c^	
PPV for biopsy recommended, %	23.71 (20.76 to 27.07)		25.37 (22.14 to 29.08)	1.66 (−1.78 to 5.11)	
PPV for biopsy performed, %	26.78 (23.43 to 30.60)		27.25 (23.90 to 31.07)	0.47 (−3.45 to 4.39)	
Sensitivity, %	86.50 (83.89 to 89.18)		87.32 (83.14 to 91.70)	0.82 (−4.35 to 6.00)	
Specificity, %	89.87 (88.90 to 90.84)		90.88 (89.42 to 92.38)	1.02 (−0.43 to 2.46)	

Abbreviations: DBT, digital breast tomosynthesis; DCIS, ductal carcinoma in situ; DM, digital mammography; PPV, positive predictive value.

^a^ Models are adjusted for age group, breast cancer family history category, race and ethnicity, history of benign breast biopsy results, breast density, screening interval, screening round, Breast Cancer Surveillance Consortium participating registry, and an interaction between Breast Cancer Surveillance Consortium participating registry and breast cancer family history category.

^b^ Absolute risk difference is significant with a false discovery rate threshold of .05 after adjusting for multiple comparisons (using the Benjamini-Hochberg procedure).

^c^ Estimates with corresponding 95% CIs of the absolute risk difference not covering 0, but not significant with a false discovery rate threshold of .05 after adjusting for multiple comparisons (using the Benjamini-Hochberg procedure).

For women with almost entirely fatty breasts, detection rate of ductal carcinoma in situ (DCIS) was significantly lower for DBT than DM (ARD, −0.71 per 1000 examinations; 95% CI, −1.03 to −0.38 per 1000 examinations) (eTable 3 in Supplement 1). For women with scattered fibroglandular densities, recall rate was significantly lower (ARD, −1.90%; 95% CI, −2.88% to −0.92%) and specificity significantly higher (ARD, 1.93%; 95% CI, 0.97%-2.89%) for DBT vs DM. PPV for recall for DBT vs DM was significantly higher for women with heterogeneously dense breasts (ARD, 1.75%; 95% CI, 0.84%-2.67%). For women with extremely dense breasts, biopsy rate was significantly higher (ARD, 0.48%; 95% CI, 0.16%-0.80%), while total ACR was significantly lower (ARD, −0.61 per 1000 examinations; 95% CI, −1.02 to −0.20 per 1000 examinations) for DBT vs DM. Although interval ACR also reduced (ARD, −0.28 per 1000 examinations; 95% CI, −0.54 to −0.03 per 1000 examinations) in this subgroup, it was not significant after further adjusting for multiple comparison. No significant ARDs were found by density for other measures.

Stratified analyses for age group, screening interval, and screening round are shown in eTables 4 to 6 in Supplement 1. For age groups, recall rates were significantly lower for DBT vs DM in groups younger than 40 years (ARD, −4.65%; 95% CI, −7.71% to −1.60%) and 60 to 69 years (−1.60%; 95% CI, −2.56% to −0.65%), and specificity was significantly higher for DBT in those age groups. PPV for recall for DBT was significantly higher in those aged 60 to 69 years (3.12%; 95% CI, 1.25%-4.98%) but not in other groups. For screening intervals, recall rates were significantly lower for DBT vs DM in annual (−1.40%; 95% CI, −2.16% to −0.64%) and triennial or longer screening intervals (−2.71%; 95% CI, −4.24% to −1.18%). Specificity was significantly higher in all screening intervals except the first screens (no previous examination). For screening rounds, recall rate for DBT vs DM was significantly lower in incident/subsequent screening round (−1.31%; 95% CI, −2.16% to −0.46%), while PPV for recall (1.76%; 95% CI, 0.83%-2.70%) and specificity (1.38%; 95% CI, 0.54%-2.22%) were significantly higher. No other significant ARDs were found.

### Cancer Characteristics

Compared to DM, DBT screen-detected cancers were more likely to be invasive (DBT: 78.8% vs DM: 74.8%) than DCIS (Table 4). Among interval cancers, proportion of invasive cancers was slightly lower for DBT (88.2%) vs DM (89.1%). Screen-detected and interval cancers with DBT vs DM were less frequently categorized by worse prognostic characteristics: *American Joint Committee on Cancer (AJCC) Staging Manual, eighth edition,* anatomic stage IIB to IV (screen-detected for DBT vs DM: 6.3% vs 8.3%), pathologic stage II to IV (screen-detected for DBT vs DM: 5.2% vs 6.4%), and axillary node positivity (screen-detected for DBT vs DM: 15.9% vs 20.4%; interval for DBT vs DM: 33.0% vs 33.4%). Small invasive tumors (≤10 mm) were more frequently diagnosed with DBT vs DM among both screen-detected (43.5% vs 39.8%, respectively) and interval cancers (27.7% vs 22.2%, respectively). Additionally, DBT (vs DM) screen-detected invasive cancers more frequently had favorable prognostic characteristics: estrogen receptor–positive or progesterone receptor–positive (91.6% vs 89.4%, respectively) and *ERBB2*-negative (91.8% vs 89.6%, respectively). Interval cancers with DBT (vs DM) less frequently had favorable characteristics: estrogen receptor–positive or progesterone receptor–positive (72.3% vs 84.8%, respectively) and *ERBB2*-negative (79.2% vs 86.2%, respectively).

**Table 4.** Characteristics of Screen-Detected and Interval Cancers by Modality^a^

Cancer characteristic	Cancer, No. (%)			
	Screen-detected cancer (n = 2641)		Interval cancer (n = 459)	
**All cancers (n = 3100)**				
	DM (n = 1942)	DBT (n = 699)	DM (n = 339)	DBT (n = 120)
Histologic type				
Missing	0	0	0	1
DCIS	489 (25.2)	148 (21.2)	37 (10.9)	14 (11.8)
Invasive	1453 (74.8)	551 (78.8)	302 (89.1)	105 (88.2)
*AJCC* anatomic stage				
Missing	44	35	9	3
0	489 (25.8)	148 (22.3)	37 (11.2)	14 (12.0)
I	982 (51.7)	391 (58.9)	143 (43.3)	56 (47.9)
IIA	270 (14.2)	83 (12.5)	72 (21.8)	18 (15.4)
IIB	93 (4.9)	26 (3.9)	42 (12.7)	15 (12.8)
III-IV	64 (3.4)	16 (2.4)	36 (10.9)	14 (12.0)
*AJCC* pathologic prognostic stage				
Missing	179	103	71	21
0	489 (27.7)	148 (24.8)	37 (13.8)	14 (14.1)
I	1161 (65.9)	417 (70.0)	188 (70.1)	69 (69.7)
IIA	58 (3.3)	21 (3.5)	11 (4.1)	6 (6.1)
IIB	16 (0.9)	5 (0.8)	8 (3.0)	2 (2.0)
III-IV	39 (2.2)	5 (0.8)	24 (9.0)	8 (8.1)
**Invasive cancers (n = 2411)**				
	DM (n = 1453)	DBT (n = 551)	DM (n = 302)	DBT (n = 105)
Histologic grade				
Missing	39	16	15	2
1	480 (33.9)	187 (35.0)	67 (23.3)	29 (28.2)
2	599 (42.4)	226 (42.2)	122 (42.5)	39 (37.9)
3	335 (23.7)	122 (22.8)	98 (34.1)	35 (34.0)
Invasive cancer size				
Missing	43	20	13	4
<6 mm	202 (14.3)	82 (15.4)	23 (8.0)	10 (9.9)
6-10 mm	360 (25.5)	149 (28.1)	41 (14.2)	18 (17.8)
11-15 mm	370 (26.2)	148 (27.9)	65 (22.5)	19 (18.8)
16-20 mm	168 (11.9)	60 (11.3)	40 (13.8)	14 (13.9)
>20 mm	310 (22.0)	92 (17.3)	120 (41.5)	40 (39.6)
Axillary lymph node status				
Missing	36	17	6	2
Negative for metastases	1128 (79.6)	449 (84.1)	197 (66.6)	69 (67.0)
Positive for metastases	289 (20.4)	85 (15.9)	99 (33.4)	34 (33.0)
ER and PR status				
Missing	18	25	5	4
ER-positive or PR-positive	1283 (89.4)	482 (91.6)	252 (84.8)	73 (72.3)
ER-negative and PR-negative	152 (10.6)	44 (8.4)	45 (15.2)	28 (27.7)
*ERBB2* (formerly *HER2*) status				
Missing	111	53	26	9
Positive	140 (10.4)	41 (8.2)	38 (13.8)	20 (20.8)
Negative	1202 (89.6)	457 (91.8)	238 (86.2)	76 (79.2)

Abbreviations: *AJCC, American Joint Committee on Cancer Staging Manual, eighth edition*; DBT, digital breast tomosynthesis; DCIS, ductal carcinoma in situ; DM, digital mammography; ER, estrogen receptor; PR, progesterone receptor.

^a^ For parameters with missing data, percentages were calculated among records with known values.

### Sensitivity Analysis

In sensitivity analyses including only women aged 50 to 74 years (eTables 7 to 12 in Supplement 1), additional measures showed elevated estimates between DBT and DM. The higher adjusted invasive CDR for DBT vs DM reached statistical significance in overall cohort (ARD, 0.81 per 1000 examinations; 95% CI, 0.31-1.31 per 1000 examinations), and in those with annual intervals (0.81 per 1000 examinations; 95% CI, 0.32-1.30 per 1000 examinations) and incident/subsequent screening rounds (ARD, 0.82 per 1000 examinations; 95% CI, 0.35-1.29 per 1000 examinations). A higher CDR was observed in women with heterogeneously dense breasts (ARD, 2.12 per 1000 examinations; 95% CI, 0.70-3.54 per 1000 examinations), and a lower DCIS detection rate was found in women with at least 2 first-degree relatives with breast cancer (ARD, −1.57 per 1000 examinations; 95% CI, −2.50 to −0.63 per 1000 examinations). Cancer characteristics did not change substantially (eTable 13 in Supplement 1).

## Discussion

This comparative cohort study represents the largest analysis to our knowledge comparing the performance of DBT vs DM in women with a family history of breast cancer, both overall and stratified by breast cancer family history category, breast density, age group, screening interval, and screening round. From analyzing more than half a million examinations, our results demonstrated that, compared to DM screening, DBT reduced recall rate and increased specificity, suggesting that DBT may result in fewer harms for women with a family history of breast cancer. The reductions in recall and increases in specificity were most evident in women with a family history of breast cancer in 1 first-degree relative, scattered fibroglandular density, those younger than 40 years or between 60 and 69 years, and those with annual or triennial or longer screening intervals, and in subsequent examinations. Prior studies comparing DBT and DM in women with a family history of breast cancer are limited.^23,24,25,26^ Consistent with our findings, 3 studies showed lower recall for DBT vs DM,^23,24,25^ although 2 of these reported nonsignificant differences, likely due to small sample sizes,^24,25^ and another study found higher specificity for DBT.^26^

We showed that the reduction in recall with DBT vs DM was achieved without compromising cancer detection. In fact, DBT had a slightly higher (though nonsignificant) adjusted CDR in the overall cohort. Our finding is consistent with 2 studies reporting similar CDR differences, although these did not provide statistical comparisons.^23,25^ DBT’s effect on cancer detection was, however, evident in our sensitivity analysis in women aged 50 to 74 years (eTable 7 in Supplement 1) for whom DBT had significantly higher invasive CDR than DM. Our study did not find a significant improvement in screening sensitivity with DBT (vs DM) overall or in stratified analyses. This finding differs from a previous study reporting higher sensitivity for DBT,^26^ although more than 70% of that study’s participants presented with symptoms^26^ whereas our study included only screening examinations.

This is the first study, to our knowledge, to show that DBT’s performance (vs DM) varied by breast cancer family history category. DBT reduced recall and increased specificity in women with 1 first-degree relative, with a similar pattern of findings in women with at least 2 first-degree relatives, although estimates did not differ significantly (likely due to smaller sample size). However, among women with only second-degree relatives, DBT had higher rates of biopsy and false-positive biopsy recommendations. Previous information on false-positive biopsy recommendations in DBT screening is conflicting. Two recent BCSC studies reported lower false-positive biopsy recommendation rates for DBT vs DM in first screening round^27^ and in low- to average-risk women (<1.67% BCSC 5-year risk) with nondense breasts^17^; the Malmö Breast Tomosynthesis Screening Trial in Sweden, however, reported higher rates of false-positive biopsy and false-positive recall.^28^ However, these studies focused on population screening rather than on women with a family history of breast cancer, as we have done. Our findings suggest that selective recommendation of DBT in screening practice may be appropriate for women with a family history of breast cancer of at least 1 first-degree relative.

DBT’s performance in women with a family history of breast cancer also varied by breast density. DBT reduced recall rates and improved specificity in women with scattered fibroglandular densities. DBT also improved PPV for recall for heterogeneously dense breasts and reduced ACR in women with extremely dense breasts. Trade-offs of DBT included lower detection of DCIS in women with almost entirely fatty breasts and increased biopsy rate in women with extremely dense breasts. These findings suggest that DBT may be more beneficial for women with scattered fibroglandular densities and heterogeneously dense breasts, while women with almost entirely fatty breasts may not experience equivalent benefit from DBT. Women with extremely dense breasts may also derive benefit from DBT as it reduces ACR, indicating more aggressive tumors are diagnosed at an earlier stage.^17^ These findings also highlight a pitfall of oversimplification by categorizing density into dichotomous dense and nondense groups.^29,30,31^ Furthermore, DBT vs DM detected an additional 1.23 cancers per 1000 examinations in women with a family history of breast cancer and heterogeneously dense breasts, although this difference was not significant, possibly due to the small sample size in this stratum and adjustment for multiple comparisons. The sensitivity analysis that reported significantly higher CDR for DBT among women with heterogeneously dense breasts supports this possibility (eTable 9 in Supplement 1). Previous studies in broader screening populations have also demonstrated that DBT (vs DM) enhances CDR in women with heterogeneously dense breasts,^30,31^ and reduces ACR in women with extremely dense breasts and high risk of breast cancer.^17^

These stratified findings advance our understanding of how DBT screening could be tailored to individual characteristics and inform a more targeted approach. By considering factors like breast density and breast cancer family history category, DBT screening has the potential to optimize screening for women at varying risk levels. Although our study provided insights into DBT and DM screening performance in women with a family history of breast cancer, other imaging modalities like magnetic resonance imaging (MRI) and contrast-enhanced mammography (CEM) have been examined in women at high risk for breast cancer.^32,33^ MRI has higher CDR than DM in women with familial risk^34^ and superior sensitivity in women at high risk for breast cancer.^35^ There are no direct comparisons of DBT and MRI in women with a family history of breast cancer. Screening data for CEM are sparse, as it has not yet received approval by the US Food and Drug Administration for routine screening. Some studies suggested higher sensitivity and comparable specificity to DM in selected women at high risk for breast cancer,^36,37,38^ but further evaluation is needed on CEM’s role in women with a family history of breast cancer.

No significant differences in ICR were found between DBT and DM in overall or stratified analyses. Current evidence regarding the impact of DBT on interval and advanced cancers remains limited, with little knowledge from studies of women with a family history of breast cancer. A recent meta-analysis provided suggestive evidence that DBT may reduce ICR in population-based screening, reporting a nonsignificant pooled ICR difference of −2.9 per 10 000 screens, and a significant difference (ICR, −5.5; 95% CI, −9.47 to −1.54) for prospective studies comparing DBT and DM in the same timeframe and regions.^39^ Our report of statistically significant decrease in ACR associated with DBT (−0.6 per 1000 examinations) among women with extremely dense breasts aligns with a recent BCSC study in broader screening populations, indicating that DBT was not associated with a significant difference in interval invasive cancer risk, but was associated with significantly lower risk of advanced cancer among women with extremely dense breasts and at high risk of breast cancer.^17^

Our study also showed that DBT (vs DM) screening finds a higher proportion of screen-detected early-stage invasive cancers with favorable prognostic characteristics, although we found no appreciable differences in interval cancer characteristics. Furthermore, screen-detected cancers generally had higher proportions of DCIS and better prognostic features, while interval cancers were more likely to present at advanced stages and had higher proportions of less favorable features, irrespective of screening modality. Our findings regarding cancer characteristics persisted in sensitivity analyses in women aged between 50 and 74 years, reinforcing the robustness of our results and highlighting potential benefits of DBT in screen-detected cancers and acknowledging the need for continued evaluation of interval cancers.

### Limitations

This study has limitations. Despite a large study population, numbers for infrequent outcomes, including interval and advanced cancers, were limited, particularly evident in sensitivity analyses. Therefore, it may be underpowered to detect minor (but clinically relevant) differences in ICR and ACR. Additionally, the lack of significant differences in metrics like recall rates for certain subgroups may be attributed to limited statistical power due to smaller subgroup sizes. For example, the number of cases in the group with at least 2 first-degree relatives was only 6% of the number in the 1 first-degree relative group. Our cohort was restricted to examinations with complete 1-year follow-up in the cancer registry used, ensuring good cancer capture but reducing sample size and potentially limiting statistical power in subgroups. As noted, findings from smaller subgroups should be interpreted with caution due to possible reduced power in stratified analyses, which may affect the strength of conclusions drawn for these subsets. Finally, our cohort predominantly consisted of examinations from non-Hispanic White women, and we did not perform analyses by race and ethnicity, limiting generalizability to other groups.

## Conclusions

In this large cohort study of DBT vs DM screening in women with a family history of breast cancer, DBT reduced recall rates and increased specificity, particularly among those with 1 first-degree relative with breast cancer or those with scattered fibroglandular breast density. While we did not observe a significant overall improvement in CDR, we found that DBT (vs DM) improved invasive cancer detection in women aged between 50 and 74 years, and led to a reduction in ACR among women with extremely dense breasts. Our study provides new evidence to guide the use of DBT in screening women with a family history of breast cancer, suggesting that DBT may be used more generally in this population or selectively based on breast cancer family history category and for extremely dense breasts.

## Notes

Supplement 1. eMethods 1. Study population eMethods 2. Data collection eMethods 3. Definition and description of performance metrics eMethods 4. Evaluation of propensity score models eMethods 5. Sensitivity analyses eTable 1. Characteristics of women with a family history of breast cancer (N = 208 945) at the first available and eligible screening examination eTable 2. Sensitivity analysis of adjusted performance measures (95% CIs) stratified by category of family history of breast cancer when restricting to subsequent examinations eTable 3. Adjusted performance measures (95% CIs) stratified by breast density eTable 4. Adjusted performance measures (95% CIs) stratified by age group eTable 5. Adjusted performance measures (95% CIs) stratified by screening interval eTable 6. Adjusted performance measures (95% CIs) stratified by screening round eTable 7. Sensitivity analysis of adjusted performance measures (95% CIs) by modality for women aged 50-74 years eTable 8. Sensitivity analysis of adjusted performance measures (95% CI) stratified by category of family history of breast cancer for women aged 50-74 years eTable 9. Sensitivity analysis of adjusted performance measures (95% CIs) stratified by breast density for women aged 50-74 years eTable 10. Sensitivity analysis of adjusted performance measures (95% CIs) stratified by age group for women aged 50-74 years eTable 11. Sensitivity analysis of adjusted performance measures (95% CIs) stratified by screening interval for women aged 50-74 years eTable 12. Sensitivity analysis of adjusted performance measures (95% CIs) stratified by screening round for women aged 50-74 years eTable 13. Sensitivity analysis of characteristics of screen-detected and interval cancers by modality when restricting to women aged 50-74 according to age at the most recent screening prior to diagnosis

Supplement 2. Data Sharing Statement
