# Supplementary material for: Tomosynthesis vs Digital Mammography Screening in Women with a Family History of Breast Cancer
Source: JAMA Oncol. 2025 May 22;11(7):742–52. doi: 10.1001/jamaoncol.2025.1209 (PMC12100508; doi:10.1001/jamaoncol.2025.1209)
Supplement: Supplement 1. — eMethods 1. Study population eMethods 2. Data collection eMethods 3. Definition and description of performance metrics eMethods 4. Evaluation of propensity score models eMethods 5. Sensitivity analyses eTable 1. Characteristics of women with a family history of breast cancer (N = 208 945) at the first available and eligible screening examination eTable 2. Sensitivity analysis of adjusted performance measures (95% CIs) stratified by category of family history of breast cancer when restricting to subsequent examinations eTable 3. Adjusted performance measures (95% CIs) stratified by breast density eTable 4. Adjusted performance measures (95% CIs) stratified by age group eTable 5. Adjusted performance measures (95% CIs) stratified by screening interval eTable 6. Adjusted performance measures (95% CIs) stratified by screening round eTable 7. Sensitivity analysis of adjusted performance measures (95% CIs) by modality for women aged 50-74 years eTable 8. Sensitivity analysis of adjusted performance measures (95% CI) stratified by category of family history of breast cancer for women aged 50-74 years eTable 9. Sensitivity analysis of adjusted performance measures (95% CIs) stratified by breast density for women aged 50-74 years eTable 10. Sensitivity analysis of adjusted performance measures (95% CIs) stratified by age group for women aged 50-74 years eTable 11. Sensitivity analysis of adjusted performance measures (95% CIs) stratified by screening interval for women aged 50-74 years eTable 12. Sensitivity analysis of adjusted performance measures (95% CIs) stratified by screening round for women aged 50-74 years eTable 13. Sensitivity analysis of characteristics of screen-detected and interval cancers by modality when restricting to women aged 50-74 according to age at the most recent screening prior to diagnosis [file jamaoncol-e251209-s001.pdf]

Supplemental Online Content

Li T, Su Y, Lee JM, et al. Tomosynthesis vs digital mammography screening in women with a family history of breast cancer. *JAMA Oncol*. Published online May 22, 2025. doi:10.1001/jamaoncol.2025.1209

eMethods 1. Study population

eMethods 2. Data collection

eMethods 3. Definition and description of performance metrics

eMethods 4. Evaluation of propensity score models

eMethods 5. Sensitivity analyses

eTable 1. Characteristics of women with a family history of breast cancer (N = 208 945) at the first available and eligible screening examination

eTable 2. Sensitivity analysis of adjusted performance measures (95% CIs) stratified by category of family history of breast cancer when restricting to subsequent examinations

eTable 3. Adjusted performance measures (95% CIs) stratified by breast density

eTable 4. Adjusted performance measures (95% CIs) stratified by age group

eTable 5. Adjusted performance measures (95% CIs) stratified by screening interval

eTable 6. Adjusted performance measures (95% CIs) stratified by screening round

eTable 7. Sensitivity analysis of adjusted performance measures (95% CIs) by modality for women aged 50-74 years

eTable 8. Sensitivity analysis of adjusted performance measures (95% CI) stratified by category of family history of breast cancer for women aged 50-74 years

eTable 9. Sensitivity analysis of adjusted performance measures (95% CIs) stratified by breast density for women aged 50-74 years

© 2025 Li T et al. *JAMA Oncology*.

eTable 10. Sensitivity analysis of adjusted performance measures (95% CIs) stratified by age group for women aged 50-74 years

eTable 11. Sensitivity analysis of adjusted performance measures (95% CIs) stratified by screening interval for women aged 50-74 years

eTable 12. Sensitivity analysis of adjusted performance measures (95% CIs) stratified by screening round for women aged 50-74 years

eTable 13. Sensitivity analysis of characteristics of screen-detected and interval cancers by modality when restricting to women aged 50-74 according to age at the most recent screening prior to diagnosis

This supplemental material has been provided by the authors to give readers additional information about their work.

© 2025 Li T et al. *JAMA Oncology*.

eMethods 1. Study population

The study cohort included all female individuals aged 18 years or older (hereafter termed women) with a self-reported family history of breast cancer who received either DBT or DM screening between January 2011 and December 2018 (Figure 1). FHBC was defined as history of breast cancer in at least one first- or second-degree female relative. Screens from women with a personal history of breast cancer or who had undergone mastectomy were excluded. We excluded examinations with: missing Breast Imaging Reporting and Data System (BI-RADS) initial assessments,[1] performed at facilities conducting fewer than 100 DBT or DM examinations, missing cancer status, and known data quality issues using the BCSC standard definition.[2] We also excluded examinations without complete 1-year cancer ascertainment in cancer registry.

eMethods 2: Data collection

Demographic and breast health history information were obtained from women through a self-administered questionnaire at the time of screening or extracted from electronic health records.

Self-reported FHBC was categorized into three groups: at least two first-degree relatives, one first-degree relative, and second-degree relatives only (i.e., with no known first-degree relatives with a FHBC). Breast density was classified by interpreting radiologists as part of routine clinical practice using BI-RADS classification: almost entirely fatty, scattered fibroglandular density, heterogeneously dense, and extremely dense.[1,3] Screening interval, determined by the time since last screening mammogram, was categorized into: first screens (those who had no previous examinations); annual screening (having had prior mammograms within 9-18 months); biennial screening (having had prior mammograms within 19-30 months); triennial or longer screening (having had prior mammograms >30 months).[4] First/prevalent examinations were defined as the initial mammogram examination regardless of modality, and incident/subsequent examinations were examinations following any prior mammogram examination.[4]

Characteristics of cancers, including histology type, American Joint Committee on Cancer (AJCC) anatomic stage [18], AJCC pathologic prognostic stage,[18] histologic grade, size of invasive cancer, axillary lymph node status, hormone receptor status, and HER2 status, were gathered from state or regional cancer registries.

eMethods 3: Definition and description of performance metrics

Recall was defined as a positive interpretation resulting from initial assessment at screening (i.e., initial BI-RADS categories 0, 3, 4, or 5[1,3]), and recall rate was calculated by dividing the number of all positive examinations by the total number of examinations.

Biopsy rate and false-positive biopsy recommendation rate were computed by dividing the number of examinations and false-positive examinations (as of final BI-RADS assessments 4 or 5[1]) leading to biopsy, respectively, by the total number of examinations.

Cancer detection rate (CDR) was computed by dividing the number of examinations with true-positive findings by the total number of examinations, and true positive was defined as a positive exam (final BI-RADS assessments of 3, 4, or 5[4,5]) with ductal carcinoma in situ (DCIS) or invasive breast carcinoma diagnosed within the follow-up period. CDR was calculated for overall cancers, invasive carcinomas, and DCIS, respectively.

© 2025 Li T et al. *JAMA Oncology*.

Cancer rate was computed by dividing the number of examinations with a cancer diagnosis within the follow-up period by the total number of examinations.

Interval cancer rate (ICR) was calculated by dividing the number of false negatives by the total number of examinations, and false negative was defined as a negative examination (final BI-RADS 1 or 2[4,5]) with DCIS or invasive breast carcinoma diagnosed within the follow-up period. The ICR was calculated overall and for invasive carcinoma.

Advanced cancer was defined as AJCC pathologic prognostic stage II or higher, or anatomic stage IIb or higher if pathologic stage was missing.[6] Rates were calculated for overall advanced cancer, screening-detected advanced cancer, and interval advanced cancer, respectively.

Positive predictive values of recall (PPV1), biopsy recommendation (PPV2), and biopsy performed (PPV3) were defined as, among positive examinations, the proportion of those followed by cancers (true positive). Positive examinations included those with initial BI-RADS assessments 0, 3, 4, or 5 for PPV1, those with final BI-RADS assessments 4 or 5 for PPV2, and those with final BI-RADS 4 or 5 as well as undergoing biopsy for PPV3.[7]

Sensitivity was computed by dividing the number of true positive examinations with final BI-RADS assessments 3, 4, or 5 by the total number of examinations with cancers[8].

Specificity was calculated as the proportion of true negative examinations with initial BI-RADS assessments 1 or 2 among those without cancer diagnosis during the follow-up period[8].

eMethods 4: Evaluation of propensity score models

We evaluated the propensity score models first by visually comparing the weighted density curves of the propensity score in DBT versus DM groups and assessing their “common support” (i.e. overlapped areas in the distribution in Figure-SM4.1).[9] In addition, we determined whether the characteristic imbalance on potential confounders between DBT and DM was ignorable after inverse probability of treatment weighting (IPTW), using a threshold of standardized mean difference (SMD) <25%.[10] Data that had a propensity score outside the “common support” were excluded from the weighted comparative analyses on performance measures. As shown in Figure-SM4.2, the distribution of the propensity score after IPTW are similar between DBT and DM. In addition, the SMD on potential confounders between DBT and DM are less than 25% as shown in Table-SM4.

eMethods 5: Sensitivity analyses

Sensitivity analyses of all the performance outcomes and cancer characteristics were conducted restricting to examinations occurring at age 50-74 years to reflect the common target group in organized screening programs in Oceania and Europe.[11]

© 2025 Li T et al. *JAMA Oncology*.

Figure-SM4.1: Distribution of propensity score by modality (unweighted)

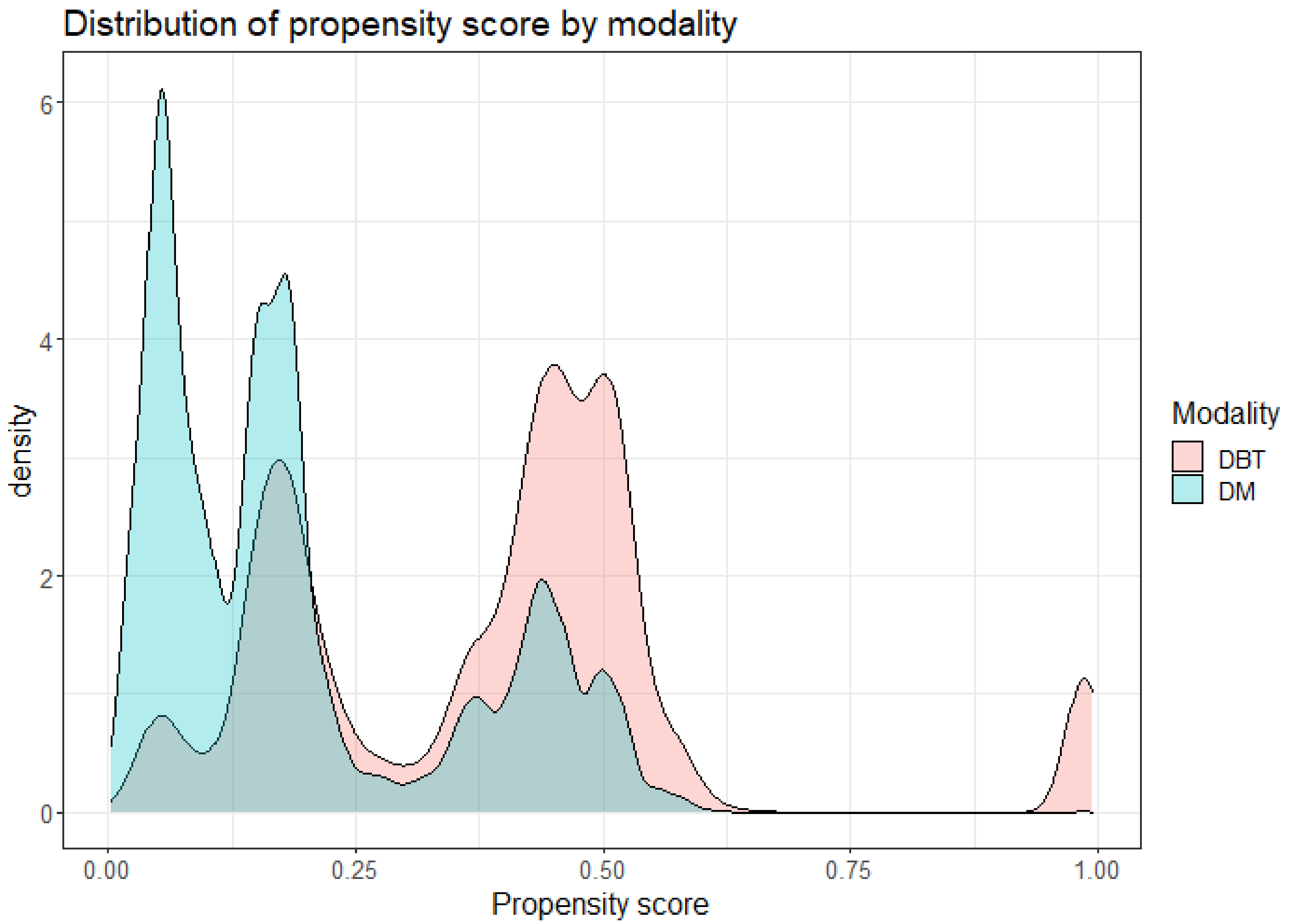

© 2025 Li T et al. *JAMA Oncology*.

Figure-SM4.2: Distribution of propensity score by modality (IPTW-weighted)

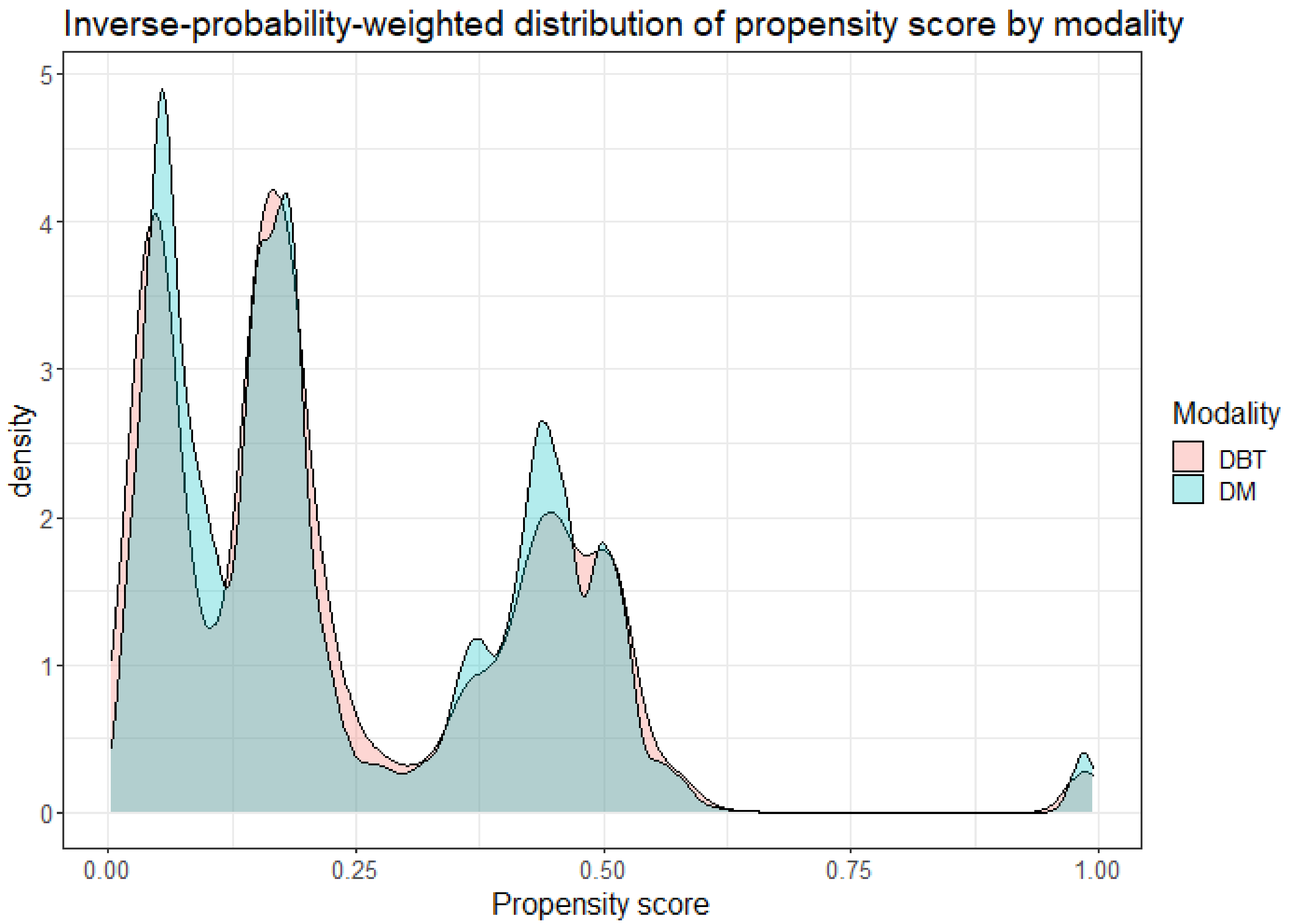

© 2025 Li T et al. *JAMA Oncology*.

Table-SM4: Standardized mean difference (%) on potential confounders between DBT vs DM examinations after IPTW in overall cohort of women with a FHBC and stratified by category of FHBC, breast density, age group, screening interval and screening round.

	Overall	Stratified by family history of breast cancer			Stratified by BI-RADS breast density				Stratified by age group (years)						Stratified by screening interval				Stratified by screening round	
		At least two 1st degree	One 1st degree	2nd degree only, with no known 1st degree	Almost entirely fatty	Scattered fibroglandular density	Heterogeneously dense	Extremely dense	<40	40-49	50-59	60-69	70-74	>74	Annual (9-18 months)	Biennial (19-30 months)	Triennial or longer (>30 months)	First screens (no previous examination)	First/prevalent	Incident/ subsequent
**Age group**																				
<40 years	1.16	4.97	0.93	0.91	1.78	-1.45	1.23	-3.21	--	--	--	--	--	--	0.87	-0.26	-1.5	5.37	5.37	0.66
40-49 years	-1.12	-4.79	0.13	0.74	-1.57	2.11	-1.5	-2.77	--	--	--	--	--	--	-0.95	-5.34	-2.37	1.7	1.7	-1.34
50-59 years	1.51	-1.81	2.48	0.98	1.28	-1.66	2.33	3.14	--	--	--	--	--	--	1.36	2.06	2.9	-4.31	-4.31	1.73
60-69 years	-0.44	2.12	-0.48	-2.23	4.81	0.38	-0.19	2.68	--	--	--	--	--	--	-1.03	4.09	0.39	-4.91	-4.91	-0.29
70-74 years	-1.31	-1.38	-2.1	-1.03	-0.33	0.01	-2.31	2.59	--	--	--	--	--	--	-0.96	-1.76	-0.42	0.54	0.54	-1.33
≥75 years	0.5	2.78	-1.62	1.09	-7.16	-0.02	0.08	-2.94	--	--	--	--	--	--	1.29	-0.04	-0.46	-0.24	-0.24	0.56
**Family history of breast cancer**																				
2nd degree only, with no known 1st degree relative	-8.59	--	--	--	5.55	10.28	-6.82	9.65	11.18	-8.95	9.67	-9	5.92	-4.72	-7.58	-11.71	-9.35	-11.53	-11.53	-8.43
One 1st degree relative	7.21	--	--	--	-2.03	-8.14	6.74	-8.69	-8.7	8.8	-8.59	7.07	-4.28	0.6	6.41	9.52	7.57	10.28	10.28	7.04
At least two 1st degree relatives	3.4	--	--	--	-7.27	-4.98	0.09	-3.15	-8.35	0.73	-2.83	4.04	-3.08	6.6	2.68	5.75	5.01	7.56	7.56	3.33
**Ethnicity and race**																				
Asian or Pacific Islander	0.41	-3.84	-1.7	3.86	3	-1.15	-0.77	4.69	-7.63	-0.65	1.65	4.32	0.94	-2.71	-1.56	8.62	-0.74	5.58	5.58	0.13
Black	-5.12	12.22	-3.28	-8.29	3.78	6.37	-7.8	7.93	4.4	-7.45	3.77	-2.07	7.26	-11.47	-6.86	0.59	2.73	-12.61	-12.61	-4.84
Hispanic or Latina	5.82	8.47	4.68	7.06	-4.15	-3.3	3.66	-2.38	-10.66	8.42	-5	4.84	-0.79	5.75	2.63	11.39	10.3	3.34	3.34	6.05
White	-0.07	-14.02	0.41	0.45	-1.77	-2.72	3.33	-3.34	11.97	-1.35	-0.37	-2.77	-6.46	8.42	3.99	-11.51	-9.42	5.95	5.95	-0.3
Other/Unknown	2.18	7.67	1.8	1.21	0.1	-1	2.61	-7.41	-9.99	5	-1.94	0.35	1.1	-0.94	2.22	0.6	4.69	-1.26	-1.26	2.36
**Prior benign breast biopsy**																				
Yes	0.55	-0.63	0.67	-0.5	0.57	-0.31	2.12	-3.63	2.88	2.94	-1.57	-2.57	-0.85	1.23	1.21	-3.78	-1.76	-2.35	-2.35	0.55
No	1.28	12.66	3.98	-4.07	-8.25	-3.6	-3.52	2.16	-2.45	-3.62	0.79	5.38	-2.79	4.54	0.27	6.26	5.9	-4.37	-4.37	1.46
Missing	-1.98	-14.77	-5.27	5.25	9.47	4.56	2.43	0.58	0.54	1.95	0.42	-4.2	3.74	-5.86	-1.36	-4.46	-5.66	4.89	4.89	-2.2
**BI-RADS Breast density**																				

© 2025 Li T et al. *JAMA Oncology*.

Almost entirely fatty	1.91	7.88	0.98	1.78	--	--	--	--	3.71	2.79	-0.47	-0.03	-4.6	9.25	1.51	2.75	-0.45	5.52	5.52	1.74
Scattered fibroglandular density	-3.42	5.3	-1.57	-7.17	--	--	--	--	-0.24	-4.42	3.18	-3.75	-0.54	-5.91	-2.32	-3.61	0.95	-0.18	-0.18	-3.54
Heterogeneously dense	-0.64	- 11.93	-0.86	1.42	--	--	--	--	0.79	-1.05	-0.23	-0.31	3.41	-1.34	0.93	-6.19	-3.84	-4.32	-4.32	-0.5
Extremely dense	-1.89	-2.19	-2.18	-1.12	--	--	--	--	1.75	-0.62	3.76	-2.48	2.83	1.29	-1.25	-4.94	-4.32	-2.81	-2.81	-1.87
Missing	14.1	3.72	10.84	18.81	--	--	--	--	-11.03	15.28	-15.4	17.03	-1.37	1.6	7.07	23.55	14.27	10.25	10.25	14.38
**Screening interval (time since last examination)**																				
First screens (no previous examination)	-0.47	5.11	-0.15	0.34	-2.15	-0.32	-1.53	0.87	-1.09	0.61	2.31	-2.22	-0.71	-0.58	--	--	--	--	--	--
Annual (9-18 months)	0.29	-3.64	0.28	-1.66	1.58	-2.34	3.42	-3.64	-0.7	0.42	0.02	-1.21	-3.22	5.09	--	--	--	--	--	0.13
Biennial (19-30 months)	1.88	4.26	2.03	1.87	-2.86	-1.69	-0.96	2.44	1.37	-1.01	-2.24	4.76	-0.9	1.01	--	--	--	--	--	1.89
Triennial or longer (>30 months)	-0.09	2.78	0.21	-0.07	2.4	-1.44	-1.4	2.85	5.45	-0.7	-0.49	0.33	-0.61	-0.92	--	--	--	--	--	-0.13
Missing	-3.4	-5.67	-4.65	0.39	-0.04	10.87	-2.98	-0.95	-4.97	0.8	2.9	-4.83	12.33	-14.88	--	--	--	--	--	-3.68
**Screening round**																				
First/prevalent	-0.47	5.11	-0.15	0.34	-2.15	-0.32	-1.53	0.87	-1.09	0.61	2.31	-2.22	-0.71	-0.58	--	--	--	--	--	--
Incident/ subsequent	0.49	-3.9	0.15	-0.26	1.9	-0.07	1.21	-0.24	2.11	-1.07	-2.24	2.5	-1.18	2.35	--	--	--	--	--	--
Missing	-0.15	-1.47	-0.02	-0.31	0.55	1.08	1.15	-3.28	-3.87	1.9	0.13	-1.16	4.51	-4.23	--	--	--	--	--	--

References:

1. Sickles EA. ACR BI-RADS® Atlas, Breast imaging reporting and data system. *American College of Radiology*. 2013:39.
2. Breast Cancer Surveillance Consortium. BCSC Standard Definitions v3.1 (revised 2023; last updated 9/15/2023). Breast Cancer Surveillance Consortium. Accessed 5 July, 2024. https://www.bcsc-research.org/data/bcsc_standard_definitions
3. Sickles EA, D'Orsi CJ, Bassett LW, Appleton CM, Berg WA, Burnside ES. ACR BI-RAD® mammography. *ACR BI-RADS® atlas, breast imaging reporting and data system*. 2013;5:2013.
4. Kerlikowske K, Su Y-R, Sprague BL, et al. Association of Screening With Digital Breast Tomosynthesis vs Digital Mammography With Risk of Interval Invasive and Advanced Breast Cancer. *JAMA*. 2022;327(22):2220-2230. doi:10.1001/jama.2022.7672

© 2025 Li T et al. *JAMA Oncology*.

5. Sprague BL, Miglioretti DL, Lee CI, Perry H, Tosteson AAN, Kerlikowske K. New mammography screening performance metrics based on the entire screening episode. *Cancer*. Jul 15 2020;126(14):3289-3296. doi:10.1002/cncr.32939
6. Kerlikowske K, Bissell MCS, Sprague BL, et al. Advanced Breast Cancer Definitions by Staging System Examined in the Breast Cancer Surveillance Consortium. *JNCI: Journal of the National Cancer Institute*. 2021;113(7):909-916. doi:10.1093/jnci/djaa176
7. Lee CI, Abraham L, Miglioretti DL, et al. National performance benchmarks for screening digital breast tomosynthesis: update from the Breast Cancer Surveillance Consortium. *Radiology*. 2023;307(4):e222499.
8. Sprague BL, Coley RY, Lowry KP, et al. Digital Breast Tomosynthesis versus Digital Mammography Screening Performance on Successive Screening Rounds from the Breast Cancer Surveillance Consortium. *Radiology*. 2023/06/01 2023;307(5):e223142. doi:10.1148/radiol.223142
9. Garrido MM, Kelley AS, Paris J, et al. Methods for Constructing and Assessing Propensity Scores. *Health Services Research*. 2014;49(5):1701-1720. doi:https://doi.org/10.1111/1475-6773.12182
10. Stuart EA, Lee BK, Leacy FP. Prognostic score–based balance measures can be a useful diagnostic for propensity score methods in comparative effectiveness research. *Journal of Clinical Epidemiology*. 2013;66(8):S84-S90.e1. doi:10.1016/j.jclinepi.2013.01.013
11. Ren W, Chen M, Qiao Y, Zhao F. Global guidelines for breast cancer screening: A systematic review. *The Breast*. 2022/08/01/ 2022;64:85-99. doi:https://doi.org/10.1016/j.breast.2022.04.003

© 2025 Li T et al. *JAMA Oncology*.

eTable 1: Characteristics of women with a family history of breast cancer (N=208,945) at the first available and eligible screening examination.

Characteristic	Number (%)
**Median age (IQR)**	55 (47-64)
**Age group**	
<40 years	9390 (4.5%)
40-49 years	60223 (28.8%)
50-59 years	63936 (30.6%)
60-69 years	47781 (22.9%)
70-74 years	13305 (6.4%)
≥75 years	14310 (6.8%)
**Mammography modality**	
DM	179555 (85.9%)
DBT	29390 (14.1%)
**Family history of breast cancer**	
At least two 1st degree	5221 (2.5%)
One 1st degree	103425 (49.5%)
2nd degree only, with no known 1st degree	100299 (48.0%)
**Race/ethnicity**	
Asian or Pacific Islander	10621 (5.1%)
Black	30048 (14.4%)
Hispanic or Latina	11160 (5.3%)
White	149520 (71.6%)
Other/Unknown	7596 (3.6%)
**Prior benign breast biopsy**	
Yes	16586 (7.9%)
No	159713 (76.4%)
Missing	32646 (15.6%)
**BI-RADS breast density**	
Almost entirely fat	19183 (9.2%)
Scattered fibroglandular	88196 (42.2%)
Heterogeneously dense	80664 (38.6%)
Extremely dense	18128 (8.7%)
Missing	2774 (1.3%)
**Exam year**	
2011-2012	105758 (50.6%)
2013-2014	66167 (31.7%)

© 2025 Li T et al. *JAMA Oncology*.

2015-2016	20018 (9.6%)
2017-2018	17002 (8.1%)
**Screening interval (time since last examination)**	
First screens (no previous examination)	25313 (12.1%)
Annual (9-18 months)	99205 (47.5%)
Biennial (19-30 months)	29870 (14.3%)
Triennial or longer (>30 months)	35077 (16.8%)
Missing	19480 (9.3%)
**Screening round**	
First/prevalent	25313 (12.1%)
Incident/subsequent	181707 (87.0%)
Missing	1925 (0.9%)
**Academic Facility**	
No	178474 (85.4%)
Yes	30471 (14.6%)

© 2025 Li T et al. *JAMA Oncology*.

eTable 2: Sensitivity analysis of adjusted performance measures (95% confidence interval) stratified by category of family history of breast cancer when restricting to subsequent examinations

Performance measures	At least two 1st degree (N=16,244)			One 1st degree (N=259,407)			2nd degree only, with no known 1st degree (N=199,372)		
	DM screening	DBT screening	Absolute risk difference	DM screening	DBT screening	Absolute risk difference	DM screening	DBT screening	Absolute risk difference
Recall rate (%)	9.25 (8.11, 10.55)	8.12 (6.62, 9.95)	-1.13 (-2.81, 0.55)	**9.13 (8.30, 10.03)**	**7.60 (6.74, 8.56)**	**-1.53 (-2.45, -0.60)**	9.36 (8.55, 10.25)	8.62 (7.37, 10.09)	-0.74 (-2.06, 0.57)
Biopsy rate (%)	1.29 (1.05, 1.59)	1.34 (1.09, 1.65)	0.05 (-0.31, 0.41)	1.37 (1.19, 1.58)	1.50 (1.26, 1.78)	0.13 (-0.13, 0.39)	**1.17 (1.00, 1.37)**	**1.52 (1.30, 1.78)**	**0.35 (0.13, 0.56)**
False-positive biopsy recommendation rate (%)	0.73 (0.55, 0.96)	0.81 (0.58, 1.14)	0.09 (-0.23, 0.40)	0.86 (0.72, 1.03)	0.93 (0.74, 1.17)	0.07 (-0.14, 0.28)	**0.84 (0.69, 1.01)**	**1.06 (0.89, 1.27)**	**0.23 (0.05, 0.40)**
Cancer detection rate (per 1,000 exams)	6.72 (5.13, 8.78)	5.90 (3.60, 9.67)	-0.82 (-3.84, 2.21)	5.82 (5.17, 6.55)	6.34 (5.40, 7.45)	0.52 (-0.45, 1.50)	*3.92 (3.39, 4.54)*	*5.00 (4.31, 5.79)*	*1.08 (0.32, 1.84)*
Invasive cancer detection rate (per 1,000 exams)	4.86 (3.58, 6.59)	5.25 (3.23, 8.54)	0.40 (-2.31, 3.10)	4.50 (3.98, 5.09)	5.05 (4.39, 5.81)	0.55 (-0.15, 1.25)	*2.96 (2.58, 3.39)*	*3.58 (3.02, 4.23)*	*0.62 (0.03, 1.21)*
DCIS detection rate (per 1,000 exams)	*1.86 (1.26, 2.75)*	*0.65 (0.21, 2.01)*	*-1.21 (-2.10, -0.32)*	1.32 (1.08, 1.62)	1.30 (0.89, 1.88)	-0.03 (-0.56, 0.51)	0.96 (0.74, 1.25)	1.42 (0.95, 2.12)	0.46 (-0.10, 1.01)
Cancer rate (per 1,000 exams)	8.11 (6.10, 10.79)	8.38 (6.28, 11.19)	0.27 (-2.55, 3.09)	7.02 (6.18, 7.99)	7.44 (6.46, 8.58)	0.42 (-0.72, 1.56)	*4.56 (3.95, 5.26)*	*5.79 (5.08, 6.61)*	*1.23 (0.45, 2.01)*
Interval cancer rate (per 1,000 exams)	1.40 (0.76, 2.58)	2.48 (1.24, 4.98)	1.09 (-0.77, 2.94)	1.21 (0.89, 1.63)	1.10 (0.84, 1.45)	-0.10 (-0.58, 0.37)	0.64 (0.50, 0.82)	0.80 (0.56, 1.13)	0.16 (-0.15, 0.47)
Invasive interval cancer rate (per 1,000 exams)	1.40 (0.76, 2.58)	0.57 (0.22, 1.53)	-0.82 (-1.93, 0.29)	1.10 (0.80, 1.52)	0.94 (0.73, 1.22)	-0.16 (-0.59, 0.27)	0.57 (0.44, 0.74)	0.76 (0.53, 1.08)	0.19 (-0.12, 0.49)
Total advanced cancer rate (per 1,000 exams)	0.40 (0.18, 0.90)	0.72 (0.29, 1.82)	0.32 (-0.39, 1.03)	0.58 (0.47, 0.71)	0.64 (0.40, 1.02)	0.06 (-0.28, 0.40)	0.40 (0.29, 0.54)	0.40 (0.20, 0.81)	0.00 (-0.31, 0.31)
Screen-detected advanced cancer rate (per 1,000 exams)	0.21 (0.07, 0.57)	0.48 (0.15, 1.52)	0.28 (-0.31, 0.86)	0.38 (0.29, 0.50)	0.42 (0.24, 0.74)	0.04 (-0.21, 0.29)	0.29 (0.20, 0.41)	0.17 (0.07, 0.40)	-0.11 (-0.30, 0.07)
Interval advanced cancer rate (per 1,000 exams)	0.20 (0.05, 0.77)	0.24 (0.04, 1.49)	0.04 (-0.50, 0.58)	0.20 (0.14, 0.28)	0.22 (0.11, 0.41)	0.02 (-0.15, 0.19)	0.11 (0.07, 0.18)	0.23 (0.08, 0.66)	0.12 (-0.12, 0.35)
PPV1 (recall) (%)	7.37 (5.61, 9.70)	8.27 (5.42, 12.59)	0.89 (-2.53, 4.31)	*6.50 (5.79, 7.30)*	*8.54 (7.39, 9.87)*	*2.04 (0.88, 3.20)*	*4.25 (3.68, 4.90)*	*5.88 (4.91, 7.04)*	*1.63 (0.65, 2.61)*
PPV2 (biopsy recommended) (%)	36.80 (29.41, 46.04)	37.82 (24.61, 58.14)	1.03 (-13.94, 15.99)	32.45 (28.85, 36.50)	33.47 (27.95, 40.07)	1.02 (-4.75, 6.79)	25.29 (22.12, 28.92)	27.93 (24.33, 32.05)	2.64 (-1.08, 6.35)
PPV3 (biopsy performed), %	43.49 (35.68, 53.00)	39.28 (24.67, 62.56)	-4.20 (-21.97, 13.56)	37.26 (33.34, 41.64)	37.99 (32.86, 43.92)	0.73 (-4.81, 6.27)	28.68 (25.15, 32.72)	30.18 (26.54, 34.32)	1.50 (-2.70, 5.70)
Sensitivity (%)	83.42 (76.20, 91.32)	70.59 (51.80, 96.20)	-12.83 (-35.22, 9.56)	83.00 (79.11, 87.09)	85.21 (81.20, 89.41)	2.21 (-3.36, 7.77)	85.77 (82.98, 88.66)	86.24 (81.70, 91.03)	0.47 (-5.09, 6.03)
Specificity (%)	91.37 (90.17, 92.58)	92.49 (90.82, 94.20)	1.13 (-0.58, 2.83)	**91.41 (90.57, 92.26)**	**93.00 (92.14, 93.87)**	**1.59 (0.70, 2.48)**	90.99 (90.16, 91.84)	91.84 (90.51, 93.19)	0.84 (-0.45, 2.14)

© 2025 Li T et al. *JAMA Oncology*.

Bold: absolute risk difference is significant with false discovery rate threshold of 0.05 after adjusting for multiple comparison (using Benjamini–Hochberg procedure). *Italic*: estimates with corresponding 95% CI of the absolute risk difference not covering 0, but not significant with false discovery rate threshold of 0.05 after adjusting for multiple comparison (using Benjamini–Hochberg procedure).
Models are adjusted for age group, category of FHBC, ethnicity and race, history of benign breast biopsy, breast density, screening interval, screening round, BCSC participating registry, and an interaction between BCSC participating registry and category of FHBC.
Data in parenthesis are 95% confidence intervals. DBT: digital breast tomosynthesis, DM: digital mammography, DCIS: ductal carcinoma in situ, PPV: positive predictive values.

© 2025 Li T et al. *JAMA Oncology*.

eTable 3: Adjusted performance measures (95% confidence intervals) stratified by breast density

Performance measures	Almost entirely fatty (N=46,005)			Scattered fibroglandular (N=224,767)			Heterogeneously dense (N=189,036)			Extremely dense (N=37,905)		
	DM screening	DBT screening	Absolute risk difference	DM screening	DBT screening	Absolute risk difference	DM screening	DBT screening	Absolute risk difference	DM screening	DBT screening	Absolute risk difference
Recall rate (%)	6.38 (5.58, 7.31)	4.96 (3.71, 6.64)	-1.42 (-3.00, 0.16)	**9.34 (8.45, 10.33)**	**7.44 (6.43, 8.61)**	**-1.90 (-2.88, -0.92)**	11.44 (10.11, 12.93)	11.44 (10.11, 12.93)	-1.11 (-2.70, 0.49)	11.95 (10.52, 13.58)	11.39 (10.38, 12.49)	-0.56 (-2.23, 1.10)
Biopsy rate (%)	1.00 (0.83, 1.21)	1.48 (0.74, 2.96)	0.48 (-0.55, 1.51)	1.30 (1.14, 1.49)	1.32 (1.12, 1.56)	0.02 (-0.17, 0.21)	1.55 (1.29, 1.87)	1.55 (1.29, 1.87)	0.26 (-0.02, 0.55)	**1.75 (1.49, 2.07)**	**2.24 (1.96, 2.55)**	**0.48 (0.16, 0.80)**
False-positive biopsy recommendation rate (%)	0.62 (0.49, 0.79)	1.14 (0.45, 2.86)	0.52 (-0.52, 1.56)	0.81 (0.70, 0.94)	0.80 (0.68, 0.94)	-0.02 (-0.13, 0.10)	1.09 (0.88, 1.35)	1.09 (0.88, 1.35)	0.14 (-0.11, 0.40)	*1.32 (1.08, 1.61)*	*1.70 (1.46, 1.98)*	*0.38 (0.06, 0.70)*
Cancer detection rate (per 1,000 exams)	4.63 (3.92, 5.46)	3.75 (2.08, 6.76)	-0.88 (-3.17, 1.41)	5.64 (4.61, 6.89)	5.85 (4.55, 7.53)	0.22 (-1.28, 1.71)	*5.27 (4.47, 6.21)*	*6.50 (5.98, 7.07)*	*1.23 (0.36, 2.10)*	4.91 (4.17, 5.79)	5.87 (3.87, 8.92)	0.96 (-1.62, 3.55)
Invasive cancer detection rate (per 1,000 exams)	3.68 (3.13, 4.32)	3.51 (1.89, 6.52)	-0.17 (-2.41, 2.07)	4.52 (3.62, 5.66)	4.60 (3.62, 5.85)	0.08 (-1.13, 1.29)	*3.91 (3.24, 4.72)*	*5.13 (4.42, 5.95)*	*1.22 (0.15, 2.28)*	3.11 (2.47, 3.92)	3.22 (2.04, 5.10)	0.11 (-1.52, 1.75)
DCIS detection rate (per 1,000 exams)	**0.95 (0.70, 1.29)**	**0.24 (0.09, 0.62)**	**-0.71 (-1.03, -0.38)**	1.12 (0.90, 1.38)	1.25 (0.89, 1.75)	0.13 (-0.31, 0.57)	1.36 (1.07, 1.72)	1.37 (0.84, 2.23)	0.02 (-0.71, 0.74)	1.80 (1.39, 2.33)	2.65 (1.51, 4.64)	0.85 (-0.78, 2.48)
Cancer rate (per 1,000 exams)	4.96 (4.20, 5.86)	4.42 (2.49, 7.86)	-0.53 (-3.23, 2.16)	6.16 (5.09, 7.44)	6.49 (5.10, 8.27)	0.34 (-1.25, 1.92)	6.70 (5.57, 8.06)	7.98 (7.28, 8.74)	1.28 (-0.07, 2.63)	6.99 (6.16, 7.93)	7.58 (5.76, 9.98)	0.59 (-1.64, 2.82)
Interval cancer rate (per 1,000 exams)	0.33 (0.19, 0.57)	0.67 (0.33, 1.38)	0.34 (-0.22, 0.91)	0.52 (0.38, 0.71)	0.64 (0.43, 0.95)	0.12 (-0.16, 0.40)	1.43 (1.00, 2.06)	1.48 (1.09, 2.00)	0.05 (-0.67, 0.77)	2.08 (1.63, 2.65)	1.71 (0.61, 4.79)	-0.37 (-2.21, 1.47)
Invasive interval cancer rate (per 1,000 exams)	0.29 (0.16, 0.51)	0.67 (0.33, 1.38)	0.39 (-0.18, 0.95)	0.47 (0.34, 0.65)	0.54 (0.37, 0.79)	0.07 (-0.16, 0.31)	1.31 (0.88, 1.94)	1.20 (0.91, 1.58)	-0.11 (-0.75, 0.53)	*1.88 (1.48, 2.40)*	*1.02 (0.54, 1.90)*	*-0.87 (-1.66, -0.07)*
Total advanced cancer rate (per 1,000 exams)	0.32 (0.17, 0.61)	0.60 (0.27, 1.33)	0.28 (-0.26, 0.83)	0.43 (0.32, 0.58)	0.56 (0.35, 0.90)	0.13 (-0.17, 0.42)	0.63 (0.50, 0.79)	0.65 (0.36, 1.17)	0.02 (-0.40, 0.44)	**0.77 (0.54, 1.12)**	**0.16 (0.03, 0.83)**	**-0.61 (-1.02, -0.20)**
Screen-detected advanced cancer rate (per 1,000 exams)	0.27 (0.14, 0.53)	0.24 (0.07, 0.77)	-0.04 (-0.36, 0.28)	0.33 (0.23, 0.47)	0.40 (0.21, 0.77)	0.07 (-0.21, 0.35)	0.40 (0.30, 0.53)	0.38 (0.18, 0.80)	-0.01 (-0.33, 0.30)	*0.38 (0.21, 0.69)*	*0.05 (0.01, 0.43)*	*-0.33 (-0.58, -0.07)*

© 2025 Li T et al. *JAMA Oncology*.

Interval advanced cancer rate (per 1,000 exams)	0.04 (0.01, 0.17)	0.36 (0.12, 1.14)	0.32 (-0.11, 0.75)	0.10 (0.06, 0.18)	0.16 (0.06, 0.43)	0.06 (-0.11, 0.22)	0.23 (0.17, 0.32)	0.26 (0.14, 0.49)	0.04 (-0.15, 0.22)	*0.39 (0.26, 0.59)*	*0.11 (0.02, 0.54)*	*-0.28 (-0.54, -0.03)*
PPV1 (recall) (%)	7.32 (6.19, 8.66)	8.12 (4.63, 14.22)	0.80 (-3.70, 5.29)	6.11 (5.07, 7.37)	7.96 (6.27, 10.10)	1.84 (-0.08, 3.77)	**4.70 (4.07, 5.43)**	**6.46 (5.60, 7.44)**	**1.75 (0.84, 2.67)**	4.24 (3.57, 5.04)	5.69 (4.22, 7.68)	1.46 (-0.29, 3.20)
PPV2 (biopsy recommended) (%)	32.34 (27.63, 37.86)	21.64 (9.09, 51.52)	-10.70 (-29.32, 7.92)	32.63 (27.88, 38.17)	34.54 (29.49, 40.45)	1.91 (-3.99, 7.81)	26.36 (22.96, 30.27)	29.94 (26.31, 34.07)	3.58 (-0.98, 8.13)	22.22 (18.75, 26.32)	22.37 (15.71, 31.85)	0.15 (-9.07, 9.37)
PPV3 (biopsy performed), %	38.18 (33.27, 43.81)	22.93 (9.18, 57.26)	-15.25 (-36.10, 5.60)	37.63 (32.20, 43.99)	39.83 (33.69, 47.08)	2.20 (-5.18, 9.57)	29.84 (25.94, 34.33)	32.16 (28.20, 36.68)	2.32 (-2.92, 7.56)	24.75 (20.62, 29.72)	24.03 (15.93, 36.23)	-0.73 (-11.73, 10.28)
Sensitivity (%)	93.38 (90.16, 96.71)	85.67 (76.59, 95.83)	-7.71 (-18.40, 2.98)	91.22 (88.96, 93.54)	89.71 (86.20, 93.36)	-1.51 (-5.48, 2.46)	80.18 (77.10, 83.39)	82.23 (77.56, 87.18)	2.04 (-4.06, 8.15)	70.53 (64.25, 77.42)	76.63 (56.79, 103.39)	6.10 (-18.05, 30.24)
Specificity (%)	94.05 (93.22, 94.90)	95.42 (94.03, 96.83)	1.37 (-0.14, 2.87)	**91.18 (90.27, 92.09)**	**93.11 (92.06, 94.17)**	**1.93 (0.97, 2.89)**	89.03 (87.66, 90.42)	90.26 (89.02, 91.52)	1.23 (-0.34, 2.80)	88.48 (86.98, 90.01)	89.18 (88.16, 90.21)	0.70 (-0.90, 2.31)

Bold: absolute risk difference is significant with false discovery rate threshold of 0.05 after adjusting for multiple comparison (using Benjamini–Hochberg procedure). *Italic*: estimates with corresponding 95% CI of the absolute risk difference not covering 0, but not significant with false discovery rate threshold of 0.05 after adjusting for multiple comparison (using Benjamini–Hochberg procedure).

Models are adjusted for age group, category of FHBC, ethnicity and race, history of benign breast biopsy, breast density, screening interval, screening round, and BCSC participating registry.

Data in parenthesis are 95% confidence intervals. DBT: digital breast tomosynthesis, DM: digital mammography, ARD: absolute risk difference; DCIS: ductal carcinoma in situ, PPV: positive predictive values.

© 2025 Li T et al. *JAMA Oncology*.

eTable 4: Adjusted performance measures (95% confidence intervals) stratified by age group

Performance measures	<40 years (N=12,556)			40-49 years (N=116,708)			50-59 years (N=154,773)			60-69 years (N=133,112)			70-74 years (N=41,657)			≥75 years (N=43,453)		
	DM	DBT	ARD	DM	DBT	ARD	DM	DBT	ARD	DM	DBT	ARD	DM	DBT	ARD	DM	DBT	ARD
Recall rate (%)	**19.25 (16.91, 21.93)**	**14.60 (11.66, 18.29)**	**-4.65 (-7.71, -1.60)**	*13.63 (12.41, 14.98)*	*12.16 (10.87, 13.62)*	*-1.47 (-2.93, -0.01)*	*9.68 (8.85, 10.59)*	*8.41 (7.55, 9.36)*	*-1.27 (-2.23, -0.31)*	**8.37 (7.51, 9.31)**	**6.76 (6.01, 7.61)**	**-1.60 (-2.56, -0.65)**	*7.27 (6.51, 8.10)*	*6.14 (5.29, 7.11)*	*-1.13 (-2.13, -0.13)*	*7.55 (6.58, 8.66)*	*5.90 (5.02, 6.94)*	*-1.64 (-2.89, -0.40)*
Biopsy rate (%)	2.30 (1.79, 2.96)	1.82 (1.14, 2.89)	-0.49 (-1.38, 0.40)	*1.62 (1.39, 1.90)*	*1.95 (1.65, 2.30)*	*0.33 (0.07, 0.58)*	1.34 (1.16, 1.54)	1.53 (1.30, 1.80)	0.19 (-0.05, 0.44)	1.25 (1.08, 1.45)	1.54 (1.22, 1.94)	0.29 (-0.06, 0.64)	1.22 (1.05, 1.42)	1.53 (1.23, 1.91)	0.31 (-0.03, 0.65)	1.56 (1.04, 2.34)	1.18 (0.87, 1.60)	-0.39 (-1.08, 0.31)
False-positive biopsy recommendation rate (%)	2.12 (1.65, 2.72)	1.72 (1.04, 2.82)	-0.41 (-1.30, 0.49)	*1.33 (1.12, 1.58)*	*1.65 (1.39, 1.96)*	*0.32 (0.09, 0.56)*	0.97 (0.81, 1.15)	1.06 (0.85, 1.33)	0.10 (-0.12, 0.31)	0.71 (0.60, 0.85)	0.90 (0.66, 1.23)	0.18 (-0.09, 0.46)	0.60 (0.49, 0.74)	0.78 (0.60, 1.01)	0.18 (-0.06, 0.40)	0.56 (0.44, 0.70)	0.39 (0.26, 0.60)	-0.16 (-0.36, 0.04)
Cancer detection rate (per 1,000 exams)	2.56 (1.53, 4.28)	1.34 (0.62, 2.88)	-1.23 (-2.80, 0.35)	3.37 (2.84, 3.99)	3.36 (2.45, 4.61)	-0.01 (-1.13, 1.11)	4.24 (3.66, 4.91)	5.14 (4.05, 6.53)	0.90 (-0.32, 2.13)	6.15 (5.39, 7.02)	7.09 (5.86, 8.57)	0.94 (-0.42, 2.29)	7.15 (5.93, 8.61)	8.42 (6.35, 11.17)	1.28 (-1.09, 3.64)	11.41 (6.54, 19.92)	8.61 (6.24, 11.90)	-2.80 (-9.52, 3.92)
Invasive cancer detection rate (per 1,000 exams)	1.74 (0.84, 3.62)	1.34 (0.62, 2.88)	-0.40 (-1.93, 1.13)	2.22 (1.85, 2.67)	2.28 (1.43, 3.63)	0.06 (-1.02, 1.14)	*3.07 (2.63, 3.58)*	*4.08 (3.19, 5.20)*	*1.00 (0.04, 1.96)*	4.76 (4.15, 5.45)	5.37 (4.26, 6.76)	0.61 (-0.68, 1.89)	6.03 (4.92, 7.39)	6.91 (4.91, 9.74)	0.89 (-1.52, 3.29)	9.82 (5.12, 18.84)	7.45 (5.51, 10.08)	-2.37 (-8.93, 4.19)
DCIS detection rate (per 1,000 exams)	0.82 (0.64, 1.06)	--	--	1.15 (0.91, 1.45)	1.08 (0.71, 1.65)	-0.06 (-0.80, 0.67)	1.17 (0.92, 1.48)	1.07 (0.80, 1.42)	-0.10 (-0.64, 0.43)	1.39 (0.92, 2.10)	1.72 (0.91, 3.26)	0.33 (-0.17, 0.82)	1.12 (0.87, 1.45)	1.51 (0.62, 3.68)	0.39 (-0.70, 1.48)	1.59 (., .)	1.16 (., .)	-0.43 (-1.47, 0.61)
Cancer rate (per 1,000 exams)	3.98 (2.54, 6.22)	1.96 (0.97, 3.95)	-2.02 (-4.17, 0.13)	4.28 (3.65, 5.02)	4.99 (4.12, 6.04)	0.71 (-0.38, 1.79)	5.04 (4.39, 5.77)	5.94 (4.63, 7.61)	0.90 (-0.60, 2.40)	7.04 (6.23, 7.95)	8.02 (6.75, 9.54)	0.99 (-0.36, 2.34)	8.16 (6.82, 9.76)	9.23 (7.28, 11.70)	1.08 (-1.29, 3.44)	13.11 (6.92, 24.87)	9.36 (6.75, 12.98)	-3.75 (-12.49, 4.98)
Interval cancer rate (per 1,000 exams)	1.41 (0.65, 3.09)	0.62 (0.20, 1.94)	-0.79 (-2.23, 0.64)	*0.91 (0.72, 1.17)*	*1.63 (1.20, 2.20)*	*0.71 (0.19, 1.24)*	0.80 (0.61, 1.03)	0.79 (0.51, 1.25)	-0.00 (-0.42, 0.41)	0.88 (0.68, 1.15)	0.93 (0.56, 1.56)	0.05 (-0.49, 0.59)	1.01 (0.72, 1.42)	0.81 (0.38, 1.74)	-0.20 (-0.91, 0.51)	1.70 (0.51, 5.74)	0.75 (0.40, 1.41)	-0.95 (-3.08, 1.17)
Invasive interval cancer rate (per 1,000 exams)	1.33 (0.58, 3.08)	0.62 (0.20, 1.94)	-0.71 (-2.15, 0.73)	*0.83 (0.65, 1.05)*	*1.58 (1.16, 2.17)*	*0.75 (0.24, 1.27)*	0.68 (0.52, 0.88)	0.52 (0.30, 0.88)	-0.16 (-	0.79 (0.61, 1.04)	0.62 (0.38, 1.03)	-0.17 (-	0.95 (0.67, 1.36)	0.81 (0.38, 1.74)	-0.14 (-	1.68 (0.49, 5.76)	0.56 (0.24, 1.34)	-1.12 (-3.23, 0.99)

© 2025 Li T et al. *JAMA Oncology*.

									0.50, 0.17)			0.56, 0.22)			0.82, 0.54)			
Total advanced cancer rate (per 1,000 exams)	0.36 (0.26, 0.50)	--	--	0.45 (0.34, 0.60)	0.56 (0.32, 0.96)	0.10 (-0.35, 0.56)	0.55 (0.41, 0.74)	0.35 (0.20, 0.62)	-0.20 (-0.45, 0.05)	0.57 (0.36, 0.89)	0.46 (0.14, 1.48)	-0.10 (-0.43, 0.22)	0.62 (0.37, 1.04)	0.62 (0.28, 1.34)	-0.01 (-0.83, 0.81)	0.55 (., .)	1.64 (., .)	1.09 (-0.20, 2.38)
Screen-detected advanced cancer rate (per 1,000 exams)	0.08 (0.06, 0.12)	--	--	*0.26 (0.18, 0.37)*	*0.09 (0.05, 0.19)*	*-0.16 (-0.31, -0.02)*	0.36 (0.26, 0.50)	0.20 (0.11, 0.37)	-0.16 (-0.35, 0.03)	0.46 (0.27, 0.77)	0.42 (0.14, 1.24)	-0.03 (-0.35, 0.28)	0.46 (0.26, 0.82)	0.40 (0.16, 1.02)	-0.06 (-0.60, 0.47)	0.41 (., .)	1.29 (., .)	0.88 (-0.34, 2.10)
Interval advanced cancer rate (per 1,000 exams)	0.28 (0.19, 0.42)	--	--	0.19 (0.13, 0.28)	0.46 (0.23, 0.94)	0.27 (-0.15, 0.68)	0.19 (0.10, 0.35)	0.15 (0.05, 0.46)	-0.04 (-0.16, 0.08)	0.11 (0.05, 0.22)	0.04 (0.01, 0.24)	-0.07 (-0.15, 0.01)	0.16 (0.07, 0.39)	0.22 (0.07, 0.72)	0.06 (-0.39, 0.50)	0.14 (., .)	0.35 (., .)	0.21 (-0.24, 0.66)
PPV1 (recall) (%)	1.33 (0.85, 2.07)	0.91 (0.42, 1.97)	-0.42 (-1.30, 0.47)	2.53 (2.14, 3.00)	2.87 (2.16, 3.82)	0.34 (-0.57, 1.25)	*4.45 (3.85, 5.15)*	*6.37 (4.80, 8.46)*	*1.92 (0.20, 3.64)*	**7.50 (6.68, 8.42)**	**10.62 (8.90, 12.67)**	**3.12 (1.25, 4.98)**	*10.03 (8.29, 12.13)*	*14.07 (11.14, 17.77)*	*4.04 (0.87, 7.21)*	15.15 (9.48, 24.22)	14.99 (10.07, 22.33)	-0.16 (-8.96, 8.64)
PPV2 (biopsy recommended) (%)	7.05 (4.80, 10.37)	5.85 (2.84, 12.02)	-1.21 (-6.11, 3.69)	15.85 (13.54, 18.56)	14.95 (11.62, 19.25)	-0.90 (-5.13, 3.33)	24.04 (20.80, 27.79)	26.60 (19.89, 35.57)	2.56 (-4.50, 9.61)	36.95 (33.67, 40.56)	37.73 (31.37, 45.39)	0.78 (-5.79, 7.35)	44.26 (38.90, 50.35)	41.72 (34.77, 50.07)	-2.53 (-10.89, 5.82)	57.32 (43.53, 75.46)	60.28 (50.49, 71.97)	2.96 (-15.52, 21.44)
PPV3 (biopsy performed), %	7.77 (5.19, 11.62)	5.53 (1.64, 18.57)	-2.24 (-9.73, 5.25)	18.20 (15.50, 21.36)	15.26 (11.61, 20.07)	-2.93 (-7.61, 1.74)	27.60 (23.84, 31.96)	30.42 (23.79, 38.88)	2.81 (-4.31, 9.93)	42.78 (39.45, 46.38)	41.66 (35.25, 49.24)	-1.12 (-7.99, 5.76)	50.77 (44.57, 57.82)	49.27 (41.90, 57.94)	-1.50 (-10.89, 7.90)	64.39 (50.08, 82.78)	66.56 (57.96, 76.44)	2.18 (-15.87, 20.23)
Sensitivity (%)	62.81 (44.83, 87.99)	71.09 (49.63, 101.83)	8.28 (-28.45, 45.01)	78.95 (75.04, 83.08)	70.47 (59.53, 83.41)	-8.49 (-20.50, 3.52)	83.95 (80.29, 87.77)	87.21 (83.29, 91.31)	3.26 (-2.08, 8.60)	87.73 (84.74, 90.83)	86.71 (79.73, 94.30)	-1.02 (-9.10, 7.05)	87.42 (83.89, 91.11)	92.98 (86.95, 99.43)	5.56 (-0.81, 11.92)	89.39 (83.49, 95.71)	91.24 (85.63, 97.22)	1.85 (-7.00, 10.70)
Specificity (%)	**80.93 (78.51, 83.41)**	**85.50 (82.29, 88.84)**	**4.58 (1.56, 7.59)**	*86.66 (85.39, 87.94)*	*88.13 (86.79, 89.48)*	*1.47 (0.02, 2.92)*	*90.71 (89.85, 91.57)*	*92.08 (91.14, 93.03)*	*1.37 (0.40, 2.35)*	**92.21 (91.34, 93.08)**	**93.91 (93.15, 94.67)**	**1.70 (0.78, 2.62)**	*93.41 (92.63, 94.19)*	*94.68 (93.86, 95.50)*	*1.27 (0.35, 2.19)*	*93.51 (92.76, 94.27)*	*94.94 (93.88, 96.01)*	*1.42 (0.31, 2.54)*

Bold: absolute risk difference is significant with false discovery rate threshold of 0.05 after adjusting for multiple comparison (using Benjamini–Hochberg procedure). *Italic*: estimates with corresponding 95% CI of the absolute risk difference not covering 0, but not significant with false discovery rate threshold of 0.05 after adjusting for multiple comparison (using Benjamini–Hochberg procedure).

Models are adjusted for age group, category of FHBC, ethnicity and race, history of benign breast biopsy, breast density, screening interval, screening round, and BCSC participating registry.

Data in parenthesis are 95% confidence intervals. DBT: digital breast tomosynthesis, DM: digital mammography, ARD: absolute risk difference; DCIS: ductal carcinoma in situ, PPV: positive predictive values.

© 2025 Li T et al. *JAMA Oncology*.

eTable 5: Adjusted performance measures (95% confidence intervals) stratified by screening interval

Performance measures	Annual (9-18 months) (N=350,077)			Biennial (19-30 months) (N=62,763)			Triennial or longer (>30 months) (N=44,546)			First screens (no previous exam) (N=25,313)		
	DM screening	DBT screening	Absolute risk difference	DM screening	DBT screening	Absolute risk difference	DM screening	DBT screening	Absolute risk difference	DM screening	DBT screening	Absolute risk difference
Recall rate (%)	**8.25 (7.49, 9.08)**	**6.85 (6.21, 7.56)**	**-1.40 (-2.16, -0.64)**	*10.51 (9.61, 11.49)*	*8.97 (8.07, 9.98)*	*-1.53 (-2.61, -0.46)*	**14.52 (13.23, 15.93)**	**11.81 (10.49, 13.29)**	**-2.71 (-4.24, -1.18)**	*25.51 (23.32, 27.91)*	*20.98 (17.08, 25.76)*	*-4.54 (-8.58, -0.49)*
Biopsy rate (%)	*1.08 (0.93, 1.25)*	*1.21 (1.10, 1.34)*	*0.14 (0.00, 0.27)*	1.48 (1.27, 1.72)	1.52 (1.14, 2.04)	0.05 (-0.40, 0.50)	2.44 (2.09, 2.84)	2.78 (2.18, 3.54)	0.34 (-0.28, 0.95)	3.47 (2.94, 4.09)	3.76 (2.66, 5.32)	0.29 (-0.71, 1.30)
False-positive biopsy recommendation rate (%)	0.70 (0.58, 0.84)	0.76 (0.67, 0.86)	0.07 (-0.05, 0.18)	1.01 (0.84, 1.21)	0.92 (0.65, 1.30)	-0.09 (-0.43, 0.24)	1.62 (1.34, 1.96)	2.00 (1.50, 2.65)	0.37 (-0.14, 0.89)	2.86 (2.34, 3.48)	3.15 (2.08, 4.76)	0.29 (-0.76, 1.34)
Cancer detection rate (per 1,000 exams)	4.45 (3.93, 5.04)	5.01 (4.22, 5.94)	0.55 (-0.26, 1.37)	5.30 (4.50, 6.25)	6.51 (4.60, 9.21)	1.21 (-1.12, 3.53)	9.28 (7.96, 10.82)	9.32 (7.63, 11.39)	0.04 (-1.83, 1.91)	7.42 (6.43, 8.56)	7.19 (5.49, 9.42)	-0.22 (-2.48, 2.03)
Invasive cancer detection rate (per 1,000 exams)	3.40 (2.97, 3.89)	3.90 (3.30, 4.61)	0.50 (-0.12, 1.13)	4.15 (3.48, 4.95)	5.34 (3.97, 7.17)	1.18 (-0.54, 2.90)	7.04 (5.97, 8.31)	7.05 (5.73, 8.68)	0.01 (-1.56, 1.57)	5.11 (4.36, 5.99)	5.75 (4.19, 7.90)	0.64 (-1.40, 2.68)
DCIS detection rate (per 1,000 exams)	1.05 (0.87, 1.28)	1.10 (0.79, 1.54)	0.05 (-0.35, 0.45)	1.15 (0.87, 1.52)	1.18 (0.49, 2.81)	0.02 (-1.05, 1.10)	2.24 (1.76, 2.85)	2.27 (1.47, 3.51)	0.03 (-0.96, 1.03)	2.31 (1.81, 2.95)	1.44 (0.56, 3.73)	-0.87 (-2.37, 0.63)
Cancer rate (per 1,000 exams)	*5.38 (4.79, 6.03)*	*6.13 (5.45, 6.89)*	*0.75 (0.09, 1.41)*	5.96 (5.15, 6.91)	6.95 (4.96, 9.75)	0.99 (-1.41, 3.39)	11.11 (8.99, 13.74)	10.98 (9.20, 13.11)	-0.13 (-2.71, 2.45)	8.05 (6.80, 9.52)	7.60 (5.88, 9.84)	-0.44 (-3.05, 2.17)
Interval cancer rate (per 1,000 exams)	0.92 (0.77, 1.10)	1.12 (0.80, 1.56)	0.20 (-0.20, 0.59)	0.66 (0.45, 0.96)	0.44 (0.20, 0.97)	-0.22 (-0.69, 0.25)	1.83 (0.60, 5.60)	1.66 (0.95, 2.91)	-0.17 (-2.40, 2.06)	0.63 (0.27, 1.46)	0.41 (0.10, 1.71)	-0.22 (-1.05, 0.61)
Invasive interval cancer rate (per 1,000 exams)	0.84 (0.70, 1.00)	0.90 (0.70, 1.16)	0.06 (-0.20, 0.33)	0.61 (0.40, 0.91)	0.44 (0.20, 0.97)	-0.17 (-0.64, 0.30)	1.72 (0.52, 5.65)	1.17 (0.62, 2.21)	-0.55 (-2.71, 1.61)	0.45 (0.15, 1.39)	0.41 (0.10, 1.71)	-0.04 (-0.85, 0.77)
Total advanced cancer rate (per 1,000 exams)	0.40 (0.31, 0.50)	0.39 (0.22, 0.68)	-0.01 (-0.26, 0.24)	0.58 (0.38, 0.87)	0.63 (0.28, 1.46)	0.06 (-0.54, 0.65)	1.07 (0.75, 1.53)	1.72 (0.86, 3.44)	0.65 (-0.60, 1.90)	1.24 (0.86, 1.79)	1.00 (0.53, 1.86)	-0.24 (-1.05, 0.56)
Screen-detected advanced cancer rate (per 1,000 exams)	0.23 (0.17, 0.32)	0.22 (0.11, 0.45)	-0.01 (-0.19, 0.17)	0.39 (0.23, 0.67)	0.50 (0.20, 1.25)	0.11 (-0.41, 0.63)	0.93 (0.62, 1.38)	0.99 (0.31, 3.17)	0.06 (-1.13, 1.25)	1.01 (0.70, 1.46)	0.67 (0.23, 1.93)	-0.34 (-1.11, 0.43)
Interval advanced cancer rate (per 1,000 exams)	0.16 (0.11, 0.23)	0.16 (0.09, 0.28)	-0.00 (-0.12, 0.12)	0.19 (0.09, 0.37)	0.14 (0.03, 0.70)	-0.05 (-0.30, 0.20)	0.15 (0.06, 0.33)	0.74 (0.28, 1.94)	0.59 (-0.14, 1.32)	0.23 (0.08, 0.63)	0.33 (0.06, 1.87)	0.10 (-0.54, 0.73)

© 2025 Li T et al. *JAMA Oncology*.

PPV1 (recall) (%)	**5.52 (4.89, 6.24)**	**7.59 (6.54, 8.82)**	**2.07 (1.05, 3.08)**	5.06 (4.27, 6.01)	7.30 (5.19, 10.28)	2.24 (-0.23, 4.70)	6.45 (5.55, 7.49)	7.94 (6.54, 9.63)	1.49 (-0.05, 3.03)	2.94 (2.51, 3.44)	3.59 (2.35, 5.46)	0.64 (-1.01, 2.29)
PPV2 (biopsy recommended) (%)	30.90 (27.35, 34.91)	34.06 (29.89, 38.81)	3.16 (-1.45, 7.77)	27.69 (23.76, 32.28)	35.06 (28.42, 43.25)	7.36 (-0.05, 14.77)	29.69 (26.16, 33.70)	26.27 (20.66, 33.40)	-3.42 (-8.85, 2.01)	16.29 (13.41, 19.80)	17.17 (11.81, 24.96)	0.87 (-6.05, 7.80)
PPV3 (biopsy performed) (%)	35.46 (31.55, 39.87)	37.38 (32.61, 42.85)	1.92 (-3.47, 7.31)	31.66 (27.08, 37.02)	39.89 (31.40, 50.68)	8.22 (-1.92, 18.36)	33.57 (29.26, 38.50)	28.18 (22.40, 35.46)	-5.38 (-11.32, 0.55)	17.71 (14.27, 21.97)	16.39 (10.92, 24.58)	-1.32 (-8.53, 5.88)
Sensitivity (%)	82.38 (79.99, 84.84)	82.48 (75.29, 90.34)	0.10 (-7.69, 7.89)	89.06 (85.10, 93.21)	93.91 (89.84, 98.17)	4.85 (-1.54, 11.25)	89.29 (83.36, 95.65)	83.15 (74.05, 93.36)	-6.15 (-17.41, 5.12)	92.45 (87.11, 98.11)	92.95 (82.92, 104.19)	0.50 (-11.52, 12.52)
Specificity (%)	**92.17 (91.39, 92.95)**	**93.63 (92.98, 94.29)**	**1.47 (0.73, 2.21)**	**89.97 (89.04, 90.90)**	**91.62 (90.68, 92.57)**	**1.66 (0.62, 2.70)**	**86.27 (84.96, 87.59)**	**89.01 (87.66, 90.37)**	**2.74 (1.24, 4.25)**	*75.04 (72.77, 77.37)*	*79.62 (75.30, 84.19)*	*4.58 (0.38, 8.78)*

Bold: absolute risk difference is significant with false discovery rate threshold of 0.05 after adjusting for multiple comparison (using Benjamini–Hochberg procedure). *Italic*: estimates with corresponding 95% CI of the absolute risk difference not covering 0, but not significant with false discovery rate threshold of 0.05 after adjusting for multiple comparison (using Benjamini–Hochberg procedure).
Models are adjusted for age group, category of FHBC, ethnicity and race, history of benign breast biopsy, breast density, screening interval, screening round, and BCSC participating registry.
Data in parenthesis are 95% confidence intervals. DBT: digital breast tomosynthesis, DM: digital mammography, DCIS: ductal carcinoma in situ, PPV: positive predictive values.

© 2025 Li T et al. *JAMA Oncology*.

eTable 6: Adjusted performance measures (95% confidence intervals) stratified by screening round

Performance measures	First/prevalent (N=25,313)			Incident/subsequent (N=475,113)		
	DM screening	DBT screening	Absolute risk difference	DM screening	DBT screening	Absolute risk difference
Recall rate (%)	*25.51 (23.32, 27.91)*	*20.98 (17.08, 25.76)*	*-4.54 (-8.58, -0.49)*	**9.23 (8.43, 10.10)**	**7.92 (7.13, 8.80)**	**-1.31 (-2.16, -0.46)**
Biopsy rate (%)	3.47 (2.94, 4.09)	3.76 (2.66, 5.32)	0.29 (-0.71, 1.30)	*1.28 (1.12, 1.48)*	*1.49 (1.30, 1.71)*	*0.21 (0.03, 0.39)*
False-positive biopsy recommendation rate (%)	2.86 (2.34, 3.48)	3.15 (2.08, 4.76)	0.29 (-0.76, 1.34)	0.84 (0.71, 1.00)	0.98 (0.83, 1.16)	0.14 (-0.02, 0.29)
Cancer detection rate (per 1,000 exams)	7.42 (6.43, 8.56)	7.19 (5.49, 9.42)	-0.22 (-2.48, 2.03)	5.06 (4.50, 5.69)	5.66 (4.98, 6.43)	0.60 (-0.09, 1.29)
Invasive cancer detection rate (per 1,000 exams)	5.11 (4.36, 5.99)	5.75 (4.19, 7.90)	0.64 (-1.40, 2.68)	*3.87 (3.44, 4.34)*	*4.41 (3.92, 4.96)*	*0.54 (0.05, 1.03)*
DCIS detection rate (per 1,000 exams)	2.31 (1.81, 2.95)	1.44 (0.56, 3.73)	-0.87 (-2.37, 0.63)	1.19 (0.99, 1.44)	1.25 (0.92, 1.70)	0.06 (-0.36, 0.47)
Cancer rate (per 1,000 exams)	8.05 (6.80, 9.52)	7.60 (5.88, 9.84)	-0.44 (-3.05, 2.17)	6.04 (5.35, 6.81)	6.71 (6.05, 7.43)	0.67 (-0.07, 1.41)
Interval cancer rate (per 1,000 exams)	0.63 (0.27, 1.46)	0.41 (0.10, 1.71)	-0.22 (-1.05, 0.61)	0.98 (0.77, 1.25)	1.05 (0.83, 1.33)	0.07 (-0.27, 0.41)
Invasive interval cancer rate (per 1,000 exams)	0.45 (0.15, 1.39)	0.41 (0.10, 1.71)	-0.04 (-0.85, 0.77)	0.89 (0.69, 1.16)	0.84 (0.67, 1.05)	-0.05 (-0.35, 0.24)
Total advanced cancer rate (per 1,000 exams)	1.24 (0.86, 1.79)	1.00 (0.53, 1.86)	-0.24 (-1.05, 0.56)	0.50 (0.41, 0.60)	0.53 (0.35, 0.81)	0.03 (-0.22, 0.29)
Screen-detected advanced cancer rate (per 1,000 exams)	1.01 (0.70, 1.46)	0.67 (0.23, 1.93)	-0.34 (-1.11, 0.43)	0.34 (0.27, 0.42)	0.33 (0.19, 0.57)	-0.01 (-0.21, 0.19)
Interval advanced cancer rate (per 1,000 exams)	0.23 (0.08, 0.63)	0.33 (0.06, 1.87)	0.10 (-0.54, 0.73)	0.16 (0.12, 0.22)	0.21 (0.12, 0.35)	0.04 (-0.08, 0.16)
PPV1 (recall) (%)	2.94 (2.51, 3.44)	3.59 (2.35, 5.46)	0.64 (-1.01, 2.29)	**5.58 (4.97, 6.26)**	**7.34 (6.38, 8.45)**	**1.76 (0.83, 2.70)**
PPV2 (biopsy recommended) (%)	16.29 (13.41, 19.80)	17.17 (11.81, 24.96)	0.87 (-6.05, 7.80)	29.89 (26.71, 33.45)	30.85 (26.73, 35.61)	0.96 (-2.93, 4.86)
PPV3 (biopsy performed) (%)	17.71 (14.27, 21.97)	16.39 (10.92, 24.58)	-1.32 (-8.53, 5.88)	34.26 (30.79, 38.12)	34.29 (30.42, 38.64)	0.03 (-3.90, 3.95)
Sensitivity (%)	92.45 (87.11, 98.11)	92.95 (82.92, 104.19)	0.50 (-11.52, 12.52)	84.56 (82.47, 86.71)	84.64 (80.45, 89.05)	0.08 (-4.52, 4.69)
Specificity (%)	*75.04 (72.77, 77.37)*	*79.62 (75.30, 84.19)*	*4.58 (0.38, 8.78)*	**91.24 (90.42, 92.06)**	**92.61 (91.79, 93.44)**	**1.38 (0.54, 2.22)**

Bold: absolute risk difference is significant with false discovery rate threshold of 0.05 after adjusting for multiple comparison (using Benjamini–Hochberg procedure). *Italic*: estimates with corresponding 95% CI of the absolute risk difference not covering 0, but not significant with false discovery rate threshold of 0.05 after adjusting for multiple comparison (using Benjamini–Hochberg procedure).
Models are adjusted for age group, category of FHBC, ethnicity and race, history of benign breast biopsy, breast density, screening interval, screening round, and BCSC participating registry.
Data in parenthesis are 95% confidence intervals. DBT: digital breast tomosynthesis, DM: digital mammography, DCIS: ductal carcinoma in situ, PPV: positive predictive values.

© 2025 Li T et al. *JAMA Oncology*.

eTable 7: Sensitivity analysis of adjusted performance measures (95% confidence intervals) by modality for women aged 50-74 years old

Performance measures	DM screening (N=246,789)	DBT screening (N=82,753)	Absolute risk difference
Recall rate (%)	**8.84 (8.03, 9.73)**	**7.47 (6.75, 8.27)**	**-1.37 (-2.22, -0.51)**
Biopsy rate (%)	*1.29 (1.12, 1.47)*	*1.53 (1.30, 1.81)*	*0.25 (0.02, 0.48)*
False-positive biopsy recommendation rate (%)	0.82 (0.69, 0.97)	0.96 (0.77, 1.20)	0.14 (-0.05, 0.34)
Cancer detection rate (per 1,000 exams)	*5.38 (4.77, 6.08)*	*6.32 (5.52, 7.24)*	*0.94 (0.16, 1.71)*
Invasive cancer detection rate (per 1,000 exams)	**4.13 (3.68, 4.64)**	**4.94 (4.37, 5.57)**	**0.81 (0.31, 1.31)**
DCIS detection rate (per 1,000 exams)	1.25 (1.03, 1.52)	1.38 (1.08, 1.78)	0.13 (-0.25, 0.51)
Cancer rate (per 1,000 exams)	*6.24 (5.57, 7.00)*	*7.17 (6.35, 8.11)*	*0.93 (0.10, 1.76)*
Interval cancer rate (per 1,000 exams)	0.86 (0.71, 1.04)	0.85 (0.63, 1.15)	-0.01 (-0.30, 0.28)
Invasive interval cancer rate (per 1,000 exams)	0.76 (0.62, 0.93)	0.59 (0.45, 0.79)	-0.16 (-0.38, 0.05)
Total advanced cancer rate (per 1,000 exams)	0.57 (0.47, 0.69)	0.43 (0.27, 0.67)	-0.14 (-0.38, 0.10)
Screen-detected advanced cancer rate (per 1,000 exams)	0.41 (0.33, 0.52)	0.31 (0.19, 0.51)	-0.10 (-0.29, 0.10)
Interval advanced cancer rate (per 1,000 exams)	0.15 (0.12, 0.21)	0.11 (0.07, 0.20)	-0.04 (-0.12, 0.04)
PPV1 (recall) (%)	**6.20 (5.53, 6.95)**	**8.68 (7.51, 10.03)**	**2.48 (1.38, 3.58)**
PPV2 (biopsy recommended) (%)	31.55 (28.48, 34.96)	32.80 (27.68, 38.87)	1.25 (-3.51, 6.01)
PPV3 (biopsy performed) (%)	36.37 (32.98, 40.10)	37.23 (32.54, 42.60)	0.86 (-3.80, 5.52)
Sensitivity (%)	86.22 (84.10, 88.39)	87.95 (84.00, 92.09)	1.73 (-2.52, 5.98)
Specificity (%)	**91.66 (90.83, 92.49)**	**93.13 (92.38, 93.88)**	**1.47 (0.63, 2.31)**

Bold: absolute risk difference is significant with false discovery rate threshold of 0.05 after adjusting for multiple comparison (using Benjamini–Hochberg procedure). *Italic*: estimates with corresponding 95% CI of the absolute risk difference not covering 0, but not significant with false discovery rate threshold of 0.05 after adjusting for multiple comparison (using Benjamini–Hochberg procedure).
Models are adjusted for age group, category of FHBC, ethnicity and race, history of benign breast biopsy, breast density, screening interval, screening round, and BCSC participating registry.
Data in parenthesis are 95% confidence intervals (CI). DBT: digital breast tomosynthesis, DM: digital mammography, DCIS: ductal carcinoma in situ, PPV: positive predictive values.

eTable 8: Sensitivity analysis of adjusted performance measures (95% confidence interval) stratified by category of family history of breast cancer for women aged 50-74 years old

Performance measures	At least two 1st degree (N=11,913)			One 1st degree (N= 184,419)			2nd degree only, with no known 1st degree (N=133,145)		
	DM screening	DBT screening	Absolute risk difference	DM screening	DBT screening	Absolute risk difference	DM screening	DBT screening	Absolute risk difference
Recall rate (%)	*9.09 (7.89, 10.46)*	*7.45 (6.51, 8.52)*	*-1.64 (-2.83, -0.46)*	**8.85 (8.00, 9.77)**	**7.34 (6.45, 8.35)**	**-1.51 (-2.54, -0.47)**	8.80 (7.96, 9.74)	7.87 (6.63, 9.34)	-0.93 (-2.31, 0.45)
Biopsy rate (%)	1.31 (1.02, 1.68)	1.26 (0.95, 1.67)	-0.05 (-0.51, 0.40)	1.37 (1.19, 1.56)	1.60 (1.28, 2.01)	0.24 (-0.11, 0.58)	1.17 (0.99, 1.38)	1.43 (1.15, 1.77)	0.26 (-0.05, 0.56)
False-positive biopsy recommendation rate (%)	0.75 (0.55, 1.02)	0.87 (0.58, 1.32)	0.12 (-0.30, 0.55)	0.84 (0.71, 0.99)	0.98 (0.73, 1.31)	0.14 (-0.14, 0.41)	0.80 (0.65, 0.98)	0.90 (0.69, 1.18)	0.10 (-0.14, 0.34)
Cancer detection rate (per 1,000 exams)	6.54 (4.78, 8.94)	4.72 (2.54, 8.75)	-1.82 (-5.01, 1.37)	6.04 (5.31, 6.86)	7.04 (5.90, 8.40)	1.00 (-0.16, 2.17)	*4.36 (3.77, 5.05)*	*5.60 (4.75, 6.60)*	*1.24 (0.27, 2.20)*
Invasive cancer detection rate (per 1,000 exams)	4.54 (3.18, 6.48)	4.28 (2.28, 8.05)	-0.26 (-3.42, 2.91)	*4.61 (4.06, 5.22)*	*5.47 (4.64, 6.45)*	*0.87 (0.06, 1.68)*	*3.44 (3.00, 3.93)*	*4.26 (3.67, 4.96)*	*0.83 (0.19, 1.47)*
DCIS detection rate (per 1,000 exams)	**2.00 (1.32, 3.03)**	**0.43 (0.12, 1.63)**	**-1.57 (-2.50, -0.63)**	1.43 (1.15, 1.77)	1.57 (1.10, 2.22)	0.14 (-0.46, 0.73)	0.92 (0.70, 1.22)	1.33 (0.82, 2.15)	0.41 (-0.23, 1.04)
Cancer rate (per 1,000 exams)	7.74 (5.68, 10.55)	7.56 (5.06, 11.31)	-0.18 (-3.50, 3.15)	7.09 (6.27, 8.01)	7.87 (6.75, 9.18)	0.78 (-0.39, 1.96)	*4.92 (4.27, 5.66)*	*6.15 (5.23, 7.23)*	*1.23 (0.21, 2.25)*
Interval cancer rate (per 1,000 exams)	1.20 (0.60, 2.39)	2.84 (1.17, 6.90)	1.65 (-0.94, 4.23)	1.05 (0.86, 1.29)	0.83 (0.59, 1.16)	-0.22 (-0.56, 0.12)	0.56 (0.42, 0.75)	0.55 (0.33, 0.93)	-0.00 (-0.32, 0.32)
Invasive interval cancer rate (per 1,000 exams)	1.20 (0.60, 2.39)	0.24 (0.04, 1.42)	-0.96 (-1.97, 0.04)	0.94 (0.77, 1.15)	0.67 (0.48, 0.93)	-0.27 (-0.56, 0.02)	0.47 (0.33, 0.67)	0.51 (0.32, 0.82)	0.04 (-0.26, 0.33)
Total advanced cancer rate (per 1,000 exams)	0.31 (0.10, 0.94)	0.41 (0.11, 1.53)	0.10 (-0.38, 0.57)	0.66 (0.54, 0.81)	0.51 (0.31, 0.83)	-0.15 (-0.46, 0.15)	0.46 (0.33, 0.63)	0.32 (0.17, 0.61)	-0.13 (-0.40, 0.13)
Screen-detected advanced cancer rate (per 1,000 exams)	0.17 (0.05, 0.65)	0.41 (0.11, 1.53)	0.23 (-0.35, 0.82)	0.47 (0.36, 0.62)	0.36 (0.20, 0.66)	-0.11 (-0.37, 0.15)	0.35 (0.24, 0.50)	0.25 (0.14, 0.44)	-0.10 (-0.30, 0.09)
Interval advanced cancer rate (per 1,000 exams)	--	--	--	0.19 (0.13, 0.27)	0.15 (0.08, 0.28)	-0.04 (-0.16, 0.08)	0.11 (0.06, 0.19)	0.07 (0.02, 0.28)	-0.03 (-0.15, 0.08)
PPV1 (recall) (%)	7.35 (5.36, 10.08)	7.82 (4.79, 12.77)	0.47 (-3.51, 4.44)	**6.97 (6.24, 7.80)**	**9.76 (8.56, 11.13)**	**2.78 (1.64, 3.93)**	**5.01 (4.30, 5.84)**	**7.22 (5.85, 8.91)**	**2.21 (0.89, 3.53)**
PPV2 (biopsy recommended) (%)	35.45 (27.76, 45.28)	30.37 (16.50, 55.91)	-5.08 (-22.83, 12.67)	33.58 (30.37, 37.14)	34.18 (28.23, 41.39)	0.60 (-5.46, 6.66)	*27.86 (24.15, 32.13)*	*32.59 (27.94, 38.01)*	*4.74 (0.09, 9.38)*
PPV3 (biopsy performed) (%)	42.85 (34.24, 53.62)	30.51 (15.10, 61.66)	-12.34 (-34.08, 9.40)	38.63 (35.15, 42.45)	39.14 (33.76, 45.38)	0.51 (-5.15, 6.17)	31.83 (27.43, 36.94)	36.88 (31.95, 42.58)	5.05 (-0.62, 10.72)
Sensitivity (%)	84.78 (76.34, 94.15)	62.80 (39.40, 100.09)	-21.98 (-51.50, 7.54)	85.47 (83.10, 87.92)	89.43 (85.61, 93.42)	3.96 (-0.33, 8.25)	88.52 (85.46, 91.69)	90.98 (86.78, 95.38)	2.46 (-2.96, 7.88)
Specificity (%)	*91.51 (90.25, 92.80)*	*93.08 (92.07, 94.10)*	*1.57 (0.38, 2.76)*	**91.71 (90.86, 92.57)**	**93.33 (92.46, 94.21)**	**1.61 (0.65, 2.58)**	91.60 (90.72, 92.48)	92.65 (91.32, 94.00)	1.06 (-0.30, 2.41)

© 2025 Li T et al. *JAMA Oncology*.

Bold: absolute risk difference is significant with false discovery rate threshold of 0.05 after adjusting for multiple comparison (using Benjamini–Hochberg procedure). *Italic*: estimates with corresponding 95% CI of the absolute risk difference not covering 0, but not significant with false discovery rate threshold of 0.05 after adjusting for multiple comparison (using Benjamini–Hochberg procedure).
Models are adjusted for age group, category of FHBC, ethnicity and race, history of benign breast biopsy, breast density, screening interval, screening round, BCSC participating registry, and an interaction between BCSC participating registry and category of FHBC.
Data in parenthesis are 95% confidence intervals. DBT: digital breast tomosynthesis, DM: digital mammography, DCIS: ductal carcinoma in situ, PPV: positive predictive values.

© 2025 Li T et al. *JAMA Oncology*.

eTable 9: Sensitivity analysis of adjusted performance measures (95% confidence intervals) stratified by breast density for women aged 50-74 years old

Performance measures	Almost entirely fatty (N=34,413)			Scattered fibroglandular (N=159,887)			Heterogeneously dense (N=114,701)			Extremely dense (N=17,453)		
	DM screening	DBT screening	Absolute risk difference	DM screening	DBT screening	Absolute risk difference	DM screening	DBT screening	Absolute risk difference	DM screening	DBT screening	Absolute risk difference
Recall rate (%)	6.25 (5.39, 7.24)	4.92 (3.35, 7.23)	-1.33 (-3.35, 0.69)	**8.72 (7.85, 9.67)**	**7.07 (6.21, 8.04)**	**-1.65 (-2.51, -0.79)**	9.64 (8.51, 10.92)	8.77 (7.74, 9.94)	-0.87 (-2.29, 0.56)	9.28 (8.09, 10.65)	9.11 (8.14, 10.19)	-0.17 (-1.62, 1.28)
Biopsy rate (%)	0.99 (0.80, 1.23)	1.73 (0.77, 3.90)	0.74 (-0.67, 2.14)	1.23 (1.08, 1.40)	1.36 (1.14, 1.62)	0.13 (-0.06, 0.33)	*1.40 (1.16, 1.67)*	*1.68 (1.47, 1.92)*	*0.29 (0.01, 0.56)*	*1.49 (1.23, 1.80)*	*2.14 (1.72, 2.66)*	*0.65 (0.13, 1.17)*
False-positive biopsy recommendation rate (%)	0.62 (0.48, 0.80)	1.46 (0.54, 3.92)	0.84 (-0.60, 2.28)	0.76 (0.65, 0.89)	0.79 (0.66, 0.95)	0.03 (-0.09, 0.15)	0.91 (0.74, 1.12)	0.98 (0.79, 1.21)	0.07 (-0.17, 0.31)	1.00 (0.79, 1.27)	1.44 (1.05, 1.99)	0.45 (-0.07, 0.96)
Cancer detection rate (per 1,000 exams)	4.48 (3.70, 5.43)	3.16 (1.81, 5.50)	-1.33 (-3.27, 0.61)	5.38 (4.57, 6.32)	6.40 (4.81, 8.52)	1.02 (-0.63, 2.68)	**5.57 (4.76, 6.52)**	**7.69 (6.57, 9.00)**	**2.12 (0.70, 3.54)**	5.55 (4.33, 7.12)	6.96 (3.94, 12.28)	1.41 (-2.69, 5.51)
Invasive cancer detection rate (per 1,000 exams)	3.51 (2.92, 4.21)	2.90 (1.61, 5.25)	-0.60 (-2.49, 1.28)	4.23 (3.62, 4.94)	5.05 (3.70, 6.89)	0.82 (-0.59, 2.24)	*4.17 (3.57, 4.87)*	*6.09 (4.93, 7.51)*	*1.92 (0.46, 3.38)*	3.79 (2.83, 5.08)	3.29 (1.71, 6.35)	-0.50 (-2.98, 1.98)
DCIS detection rate (per 1,000 exams)	**0.98 (0.67, 1.42)**	**0.25 (0.08, 0.76)**	**-0.73 (-1.12, -0.33)**	1.15 (0.90, 1.45)	1.35 (1.06, 1.72)	0.20 (-0.17, 0.58)	1.41 (1.07, 1.85)	1.60 (1.08, 2.37)	0.19 (-0.53, 0.91)	1.76 (1.13, 2.75)	3.67 (1.53, 8.78)	1.91 (-1.26, 5.08)
Cancer rate (per 1,000 exams)	4.81 (3.99, 5.81)	3.65 (2.09, 6.36)	-1.17 (-3.32, 0.99)	5.95 (5.09, 6.97)	7.03 (5.37, 9.19)	1.08 (-0.63, 2.78)	*6.82 (5.97, 7.79)*	*8.81 (7.53, 10.32)*	*1.99 (0.38, 3.61)*	7.64 (6.27, 9.30)	9.45 (5.83, 15.29)	1.81 (-2.86, 6.48)
Interval cancer rate (per 1,000 exams)	0.33 (0.17, 0.64)	0.49 (0.21, 1.14)	0.16 (-0.32, 0.64)	0.58 (0.41, 0.81)	0.63 (0.37, 1.06)	0.05 (-0.31, 0.41)	1.25 (1.04, 1.50)	1.13 (0.81, 1.57)	-0.12 (-0.58, 0.34)	2.08 (1.54, 2.81)	2.48 (0.85, 7.26)	0.40 (-2.31, 3.11)
Invasive interval cancer rate (per 1,000 exams)	0.27 (0.13, 0.56)	0.49 (0.21, 1.14)	0.22 (-0.27, 0.70)	0.53 (0.37, 0.76)	0.50 (0.31, 0.82)	-0.03 (-0.32, 0.26)	1.09 (0.90, 1.33)	0.76 (0.54, 1.07)	-0.34 (-0.68, 0.01)	1.79 (1.28, 2.51)	0.97 (0.52, 1.81)	-0.82 (-1.70, 0.06)
Total advanced cancer rate (per 1,000 exams)	0.27 (0.14, 0.55)	0.39 (0.14, 1.07)	0.12 (-0.30, 0.54)	*0.52 (0.39, 0.71)*	*0.29 (0.16, 0.50)*	*-0.24 (-0.48, 0.00)*	0.70 (0.54, 0.91)	0.68 (0.36, 1.29)	-0.02 (-0.52, 0.48)	0.51 (0.22, 1.15)	0.38 (0.07, 1.90)	-0.13 (-0.90, 0.63)
Screen-detected advanced cancer rate (per 1,000 exams)	0.27 (0.14, 0.55)	0.33 (0.10, 1.05)	0.05 (-0.36, 0.46)	0.40 (0.28, 0.56)	0.22 (0.11, 0.42)	-0.18 (-0.38, 0.01)	0.47 (0.33, 0.66)	0.50 (0.22, 1.11)	0.03 (-0.44, 0.49)	0.24 (0.07, 0.80)	0.12 (0.02, 0.98)	-0.12 (-0.50, 0.26)

© 2025 Li T et al. *JAMA Oncology*.

Interval advanced cancer rate (per 1,000 exams)	--	--	--	0.12 (0.07, 0.21)	0.07 (0.02, 0.31)	-0.05 (-0.19, 0.08)	0.23 (0.16, 0.34)	0.18 (0.09, 0.37)	-0.05 (-0.21, 0.11)	0.26 (0.10, 0.67)	0.25 (0.05, 1.22)	-0.01 (-0.51, 0.50)
PPV1 (recall) (%)	7.23 (6.10, 8.56)	6.74 (3.68, 12.34)	-0.49 (-4.77, 3.78)	*6.27 (5.44, 7.24)*	*9.12 (7.06, 11.79)*	*2.85 (0.69, 5.02)*	**5.93 (5.21, 6.74)**	**8.97 (7.41, 10.86)**	**3.04 (1.42, 4.67)**	6.05 (4.93, 7.43)	9.17 (5.67, 14.84)	3.12 (-1.48, 7.72)
PPV2 (biopsy recommended) (%)	*31.53 (26.57, 37.40)*	*14.91 (5.42, 41.02)*	*-16.62 (-32.39, -0.84)*	32.96 (29.20, 37.21)	35.31 (29.09, 42.85)	2.34 (-3.72, 8.40)	*30.61 (27.45, 34.12)*	*38.58 (32.11, 46.34)*	*7.97 (0.74, 15.21)*	29.35 (23.44, 36.73)	27.19 (16.97, 43.58)	-2.15 (-16.55, 12.24)
PPV3 (biopsy performed) (%)	*37.43 (32.27, 43.41)*	*15.76 (5.41, 45.90)*	*-21.66 (-39.47, -3.86)*	38.06 (33.71, 42.96)	41.76 (34.57, 50.44)	3.70 (-3.69, 11.09)	34.83 (31.32, 38.74)	41.71 (34.94, 49.78)	6.87 (-1.16, 14.91)	33.15 (26.02, 42.24)	32.52 (19.47, 54.33)	-0.63 (-18.99, 17.73)
Sensitivity (%)	93.38 (89.29, 97.66)	88.54 (82.83, 94.64)	-4.84 (-12.80, 3.12)	90.44 (87.79, 93.16)	90.89 (86.27, 95.76)	0.45 (-4.65, 5.55)	81.89 (78.48, 85.46)	86.52 (83.18, 90.00)	4.63 (-0.27, 9.54)	72.52 (64.98, 80.94)	74.19 (54.93, 100.20)	1.67 (-21.88, 25.22)
Specificity (%)	94.18 (93.29, 95.07)	95.40 (93.53, 97.30)	1.22 (-0.78, 3.22)	**91.78 (90.91, 92.67)**	**93.53 (92.68, 94.39)**	**1.75 (0.92, 2.57)**	90.87 (89.71, 92.05)	91.94 (90.85, 93.05)	1.07 (-0.32, 2.46)	91.22 (90.01, 92.44)	91.65 (90.76, 92.54)	0.43 (-0.91, 1.78)

Bold: absolute risk difference is significant with false discovery rate threshold of 0.05 after adjusting for multiple comparison (using Benjamini–Hochberg procedure). *Italic*: estimates with corresponding 95% CI of the absolute risk difference not covering 0, but not significant with false discovery rate threshold of 0.05 after adjusting for multiple comparison (using Benjamini–Hochberg procedure).
Models are adjusted for age group, category of FHBC, ethnicity and race, history of benign breast biopsy, breast density, screening interval, screening round, and BCSC participating registry.
Data in parenthesis are 95% confidence intervals. DBT: digital breast tomosynthesis, DM: digital mammography, DCIS: ductal carcinoma in situ, PPV: positive predictive values.

© 2025 Li T et al. *JAMA Oncology*.

eTable 10: Sensitivity analysis of adjusted performance measures (95% confidence intervals) stratified by age group for women aged 50-74 years old

Performance measures	50-59 years (N=154,773)			60-69 years (N=133,112)			70-74 years (N=41,657)		
	DM screening	DBT screening	Absolute risk difference	DM screening	DBT screening	Absolute risk difference	DM screening	DBT screening	Absolute risk difference
Recall rate (%)	*9.68 (8.85, 10.59)*	*8.41 (7.55, 9.36)*	*-1.27 (-2.23, -0.31)*	**8.37 (7.51, 9.31)**	**6.76 (6.01, 7.61)**	**-1.60 (-2.56, -0.65)**	*7.27 (6.51, 8.10)*	*6.14 (5.29, 7.11)*	*-1.13 (-2.13, -0.13)*
Biopsy rate (%)	1.34 (1.16, 1.54)	1.53 (1.30, 1.80)	0.19 (-0.05, 0.44)	1.25 (1.08, 1.45)	1.54 (1.22, 1.94)	0.29 (-0.06, 0.64)	1.22 (1.05, 1.42)	1.53 (1.23, 1.91)	0.31 (-0.03, 0.65)
False-positive biopsy recommendation rate (%)	0.97 (0.81, 1.15)	1.06 (0.85, 1.33)	0.10 (-0.12, 0.31)	0.71 (0.60, 0.85)	0.90 (0.66, 1.23)	0.18 (-0.09, 0.46)	0.60 (0.49, 0.74)	0.78 (0.60, 1.01)	0.18 (-0.06, 0.40)
Cancer detection rate (per 1,000 exams)	4.24 (3.66, 4.91)	5.14 (4.05, 6.53)	0.90 (-0.32, 2.13)	6.15 (5.39, 7.02)	7.09 (5.86, 8.57)	0.94 (-0.42, 2.29)	7.15 (5.93, 8.61)	8.42 (6.35, 11.17)	1.28 (-1.09, 3.64)
Invasive cancer detection rate (per 1,000 exams)	*3.07 (2.63, 3.58)*	*4.08 (3.19, 5.20)*	*1.00 (0.04, 1.96)*	4.76 (4.15, 5.45)	5.37 (4.26, 6.76)	0.61 (-0.68, 1.89)	6.03 (4.92, 7.39)	6.91 (4.91, 9.74)	0.89 (-1.52, 3.29)
DCIS detection rate (per 1,000 exams)	1.17 (0.92, 1.48)	1.07 (0.80, 1.42)	-0.10 (-0.64, 0.43)	1.39 (0.92, 2.10)	1.72 (0.91, 3.26)	0.33 (-0.17, 0.82)	1.12 (0.87, 1.45)	1.51 (0.62, 3.68)	0.39 (-0.70, 1.48)
Cancer rate (per 1,000 exams)	5.04 (4.39, 5.77)	5.94 (4.63, 7.61)	0.90 (-0.60, 2.40)	7.04 (6.23, 7.95)	8.02 (6.75, 9.54)	0.99 (-0.36, 2.34)	8.16 (6.82, 9.76)	9.23 (7.28, 11.70)	1.08 (-1.29, 3.44)
Interval cancer rate (per 1,000 exams)	0.80 (0.61, 1.03)	0.79 (0.51, 1.25)	-0.00 (-0.42, 0.41)	0.88 (0.68, 1.15)	0.93 (0.56, 1.56)	0.05 (-0.49, 0.59)	1.01 (0.72, 1.42)	0.81 (0.38, 1.74)	-0.20 (-0.91, 0.51)
Invasive interval cancer rate (per 1,000 exams)	0.68 (0.52, 0.88)	0.52 (0.30, 0.88)	-0.16 (-0.50, 0.17)	0.79 (0.61, 1.04)	0.62 (0.38, 1.03)	-0.17 (-0.56, 0.22)	0.95 (0.67, 1.36)	0.81 (0.38, 1.74)	-0.14 (-0.82, 0.54)
Total advanced cancer rate (per 1,000 exams)	0.55 (0.41, 0.74)	0.35 (0.20, 0.62)	-0.20 (-0.45, 0.05)	0.57 (0.36, 0.89)	0.46 (0.14, 1.48)	-0.10 (-0.43, 0.22)	0.62 (0.37, 1.04)	0.62 (0.28, 1.34)	-0.01 (-0.83, 0.81)
Screen-detected advanced cancer rate (per 1,000 exams)	0.36 (0.26, 0.50)	0.20 (0.11, 0.37)	-0.16 (-0.35, 0.03)	0.46 (0.27, 0.77)	0.42 (0.14, 1.24)	-0.03 (-0.35, 0.28)	0.46 (0.26, 0.82)	0.40 (0.16, 1.02)	-0.06 (-0.60, 0.47)
Interval advanced cancer rate (per 1,000 exams)	0.19 (0.10, 0.35)	0.15 (0.05, 0.46)	-0.04 (-0.16, 0.08)	0.11 (0.05, 0.22)	0.04 (0.01, 0.24)	-0.07 (-0.15, 0.01)	0.16 (0.07, 0.39)	0.22 (0.07, 0.72)	0.06 (-0.39, 0.50)
PPV1 (recall) (%)	*4.45 (3.85, 5.15)*	*6.37 (4.80, 8.46)*	*1.92 (0.20, 3.64)*	**7.50 (6.68, 8.42)**	**10.62 (8.90, 12.67)**	**3.12 (1.25, 4.98)**	*10.03 (8.29, 12.13)*	*14.07 (11.14, 17.77)*	*4.04 (0.87, 7.21)*
PPV2 (biopsy recommended) (%)	24.04 (20.80, 27.79)	26.60 (19.89, 35.57)	2.56 (-4.50, 9.61)	36.95 (33.67, 40.56)	37.73 (31.37, 45.39)	0.78 (-5.79, 7.35)	44.26 (38.90, 50.35)	41.72 (34.77, 50.07)	-2.53 (-10.89, 5.82)
PPV3 (biopsy performed) (%)	27.60 (23.84, 31.96)	30.42 (23.79, 38.88)	2.81 (-4.31, 9.93)	42.78 (39.45, 46.38)	41.66 (35.25, 49.24)	-1.12 (-7.99, 5.76)	50.77 (44.57, 57.82)	49.27 (41.90, 57.94)	-1.50 (-10.89, 7.90)
Sensitivity (%)	83.95 (80.29, 87.77)	87.21 (83.29, 91.31)	3.26 (-2.08, 8.60)	87.73 (84.74, 90.83)	86.71 (79.73, 94.30)	-1.02 (-9.10, 7.05)	87.42 (83.89, 91.11)	92.98 (86.95, 99.43)	5.56 (-0.81, 11.92)
Specificity (%)	*90.71 (89.85, 91.57)*	*92.08 (91.14, 93.03)*	*1.37 (0.40, 2.35)*	**92.21 (91.34, 93.08)**	**93.91 (93.15, 94.67)**	**1.70 (0.78, 2.62)**	*93.41 (92.63, 94.19)*	*94.68 (93.86, 95.50)*	*1.27 (0.35, 2.19)*

© 2025 Li T et al. *JAMA Oncology*.

Bold: absolute risk difference is significant with false discovery rate threshold of 0.05 after adjusting for multiple comparison (using Benjamini–Hochberg procedure). *Italic*: estimates with corresponding 95% CI of the absolute risk difference not covering 0, but not significant with false discovery rate threshold of 0.05 after adjusting for multiple comparison (using Benjamini–Hochberg procedure).
Models are adjusted for age group, category of FHBC, ethnicity and race, history of benign breast biopsy, breast density, screening interval, screening round, and BCSC participating registry.
Data in parenthesis are 95% confidence intervals. DBT: digital breast tomosynthesis, DM: digital mammography, DCIS: ductal carcinoma in situ, PPV: positive predictive values.

© 2025 Li T et al. *JAMA Oncology*.

eTable 11: Sensitivity analysis of adjusted performance measures (95% confidence intervals) stratified by screening interval for women aged 50-74 years old

Performance measures	Annual (9-18 months) (N=240,701)			Biennial (19-30 months) (N=41,631)			Triennial or longer (>30 months) (N=27,678)			First screens (no previous exam) (N=7,619)		
	DM screening	DBT screening	Absolute risk difference	DM screening	DBT screening	Absolute risk difference	DM screening	DBT screening	Absolute risk difference	DM screening	DBT screening	Absolute risk difference
Recall rate (%)	**7.61 (6.88, 8.42)**	**6.29 (5.71, 6.94)**	**-1.32 (-2.08, -0.55)**	**9.45 (8.61, 10.38)**	**8.01 (7.18, 8.94)**	**-1.44 (-2.42, -0.47)**	**13.36 (12.19, 14.65)**	**10.79 (9.64, 12.08)**	**-2.57 (-4.00, -1.13)**	25.30 (22.47, 28.48)	20.74 (16.27, 26.43)	-4.56 (-9.39, 0.26)
Biopsy rate (%)	1.04 (0.89, 1.21)	1.19 (1.05, 1.36)	0.16 (-0.01, 0.32)	1.36 (1.15, 1.60)	1.45 (1.08, 1.94)	0.09 (-0.36, 0.54)	2.42 (2.12, 2.77)	2.57 (1.88, 3.51)	0.15 (-0.66, 0.95)	3.77 (3.09, 4.60)	5.01 (3.41, 7.37)	1.24 (-0.35, 2.83)
False-positive biopsy recommendation rate (%)	0.65 (0.54, 0.79)	0.69 (0.60, 0.81)	0.04 (-0.08, 0.17)	0.88 (0.73, 1.08)	0.77 (0.50, 1.19)	-0.11 (-0.48, 0.25)	1.49 (1.25, 1.78)	1.69 (1.12, 2.54)	0.20 (-0.50, 0.90)	2.75 (2.12, 3.57)	3.93 (2.39, 6.45)	1.18 (-0.45, 2.81)
Cancer detection rate (per 1,000 exams)	*4.54 (4.00, 5.15)*	*5.51 (4.71, 6.45)*	*0.97 (0.20, 1.75)*	5.40 (4.50, 6.47)	7.03 (5.12, 9.67)	1.64 (-0.72, 4.00)	10.55 (9.06, 12.27)	10.76 (8.62, 13.44)	0.22 (-2.06, 2.49)	11.37 (9.23, 14.00)	12.65 (8.76, 18.26)	1.28 (-3.94, 6.50)
Invasive cancer detection rate (per 1,000 exams)	**3.45 (3.05, 3.92)**	**4.27 (3.70, 4.92)**	**0.81 (0.32, 1.30)**	4.25 (3.51, 5.16)	6.03 (4.53, 8.02)	1.78 (-0.08, 3.64)	8.33 (7.07, 9.80)	7.45 (5.60, 9.90)	-0.88 (-3.05, 1.29)	8.71 (6.92, 10.98)	10.20 (7.51, 13.85)	1.49 (-2.10, 5.07)
DCIS detection rate (per 1,000 exams)	1.08 (0.87, 1.35)	1.25 (0.90, 1.72)	0.16 (-0.29, 0.61)	1.14 (0.79, 1.65)	1.00 (0.38, 2.69)	-0.14 (-1.28, 1.00)	2.22 (1.66, 2.96)	3.31 (2.03, 5.40)	1.09 (-0.49, 2.67)	2.65 (1.79, 3.92)	2.45 (0.31, 19.20)	-0.21 (-5.33, 4.91)
Cancer rate (per 1,000 exams)	*5.46 (4.85, 6.14)*	*6.44 (5.68, 7.31)*	*0.99 (0.27, 1.70)*	5.95 (5.05, 7.00)	7.48 (5.43, 10.31)	1.53 (-0.92, 3.98)	11.32 (9.71, 13.18)	12.10 (9.61, 15.24)	0.79 (-1.97, 3.54)	11.80 (9.54, 14.59)	12.65 (8.76, 18.26)	0.85 (-4.46, 6.15)
Interval cancer rate (per 1,000 exams)	0.92 (0.76, 1.12)	0.93 (0.64, 1.35)	0.01 (-0.36, 0.38)	0.55 (0.37, 0.82)	0.44 (0.17, 1.13)	-0.11 (-0.61, 0.39)	0.77 (0.49, 1.21)	1.34 (0.53, 3.42)	0.57 (-0.74, 1.88)	0.43 (., .)	--	--
Invasive interval cancer rate (per 1,000 exams)	0.83 (0.68, 1.01)	0.66 (0.51, 0.86)	-0.17 (-0.40, 0.07)	0.47 (0.29, 0.76)	0.44 (0.17, 1.13)	-0.03 (-0.55, 0.49)	0.59 (0.33, 1.05)	0.64 (0.29, 1.41)	0.05 (-0.53, 0.64)	0.29 (., .)	--	--
Total advanced cancer rate (per 1,000 exams)	0.44 (0.34, 0.58)	0.38 (0.21, 0.70)	-0.06 (-0.34, 0.21)	0.56 (0.37, 0.86)	0.46 (0.16, 1.35)	-0.10 (-0.68, 0.47)	1.13 (0.78, 1.64)	0.59 (0.26, 1.35)	-0.54 (-1.11, 0.03)	2.27 (1.44, 3.58)	1.83 (0.48, 6.92)	-0.44 (-2.99, 2.12)
Screen-detected advanced cancer rate (per 1,000 exams)	0.27 (0.18, 0.40)	0.24 (0.11, 0.53)	-0.03 (-0.26, 0.20)	0.42 (0.24, 0.73)	0.46 (0.16, 1.35)	0.04 (-0.54, 0.63)	*1.05 (0.72, 1.54)*	*0.42 (0.17, 1.05)*	*-0.63 (-1.14, -0.13)*	2.13 (1.34, 3.38)	1.83 (0.48, 6.92)	-0.29 (-2.83, 2.24)
Interval advanced cancer rate (per 1,000 exams)	0.17 (0.12, 0.25)	0.14 (0.08, 0.25)	-0.04 (-0.14, 0.07)	0.15 (0.04, 0.56)	--	--	0.08 (., .)	0.18 (., .)	0.09 (., .)	0.14 (., .)	--	--

© 2025 Li T et al. *JAMA Oncology*.

PPV1 (recall) (%)	**6.12 (5.46, 6.86)**	**9.10 (7.94, 10.44)**	**2.99 (1.98, 4.00)**	*5.74 (4.71, 6.98)*	*8.86 (6.38, 12.29)*	*3.12 (0.22, 6.03)*	*7.92 (6.83, 9.20)*	*9.97 (8.04, 12.36)*	*2.05 (0.05, 4.04)*	4.55 (3.57, 5.79)	6.10 (4.15, 8.96)	1.55 (-1.15, 4.25)
PPV2 (biopsy recommended) (%)	*32.78 (29.44, 36.51)*	*37.53 (33.34, 42.25)*	*4.75 (0.74, 8.75)*	*29.51 (25.10, 34.70)*	*39.53 (31.41, 49.74)*	*10.01 (0.60, 19.43)*	33.70 (29.63, 38.32)	30.61 (23.69, 39.55)	-3.09 (-10.02, 3.83)	23.09 (18.03, 29.57)	21.89 (14.12, 33.94)	-1.20 (-11.60, 9.21)
PPV3 (biopsy performed) (%)	37.44 (33.86, 41.40)	41.89 (37.61, 46.65)	4.45 (-0.02, 8.91)	34.82 (29.67, 40.86)	46.71 (35.22, 61.94)	11.89 (-2.17, 25.94)	38.40 (33.19, 44.42)	34.18 (25.76, 45.33)	-4.22 (-13.76, 5.31)	26.96 (20.98, 34.65)	21.58 (11.93, 39.06)	-5.38 (-18.83, 8.07)
Sensitivity (%)	82.96 (80.18, 85.83)	85.47 (79.18, 92.26)	2.51 (-4.35, 9.38)	90.74 (86.77, 94.88)	93.97 (89.33, 98.85)	3.23 (-3.65, 10.11)	92.99 (90.14, 95.92)	87.56 (77.38, 99.09)	-5.43 (-16.58, 5.73)	--	--	--
Specificity (%)	**92.82 (92.07, 93.57)**	**94.24 (93.66, 94.83)**	**1.43 (0.69, 2.16)**	**91.04 (90.16, 91.93)**	**92.65 (91.75, 93.55)**	**1.61 (0.64, 2.57)**	**87.56 (86.37, 88.76)**	**90.16 (88.99, 91.36)**	**2.61 (1.21, 4.01)**	75.57 (72.58, 78.68)	80.28 (75.42, 85.46)	4.71 (-0.17, 9.60)

Bold: absolute risk difference is significant with false discovery rate threshold of 0.05 after adjusting for multiple comparison (using Benjamini–Hochberg procedure). *Italic*: estimates with corresponding 95% CI of the absolute risk difference not covering 0, but not significant with false discovery rate threshold of 0.05 after adjusting for multiple comparison (using Benjamini–Hochberg procedure).

Models are adjusted for age group, category of FHBC, ethnicity and race, history of benign breast biopsy, breast density, screening interval, screening round, and BCSC participating registry.

Data in parenthesis are 95% confidence intervals. DBT: digital breast tomosynthesis, DM: digital mammography, DCIS: ductal carcinoma in situ, PPV: positive predictive values.

© 2025 Li T et al. *JAMA Oncology*.

eTable 12: Sensitivity analysis of adjusted performance measures (95% confidence intervals) stratified by screening round for women aged 50-74 years old

Performance measures	First/prevalent (N=7,619)			Incident/subsequent (N=321,079)		
	DM screening	DBT screening	Absolute risk difference	DM screening	DBT screening	Absolute risk difference
Recall rate (%)	25.30 (22.47, 28.48)	20.74 (16.27, 26.43)	-4.56 (-9.39, 0.26)	**8.42 (7.66, 9.24)**	**7.18 (6.46, 7.97)**	**-1.24 (-2.04, -0.44)**
Biopsy rate (%)	3.77 (3.09, 4.60)	5.01 (3.41, 7.37)	1.24 (-0.35, 2.83)	*1.22 (1.06, 1.40)*	*1.46 (1.24, 1.71)*	*0.24 (0.02, 0.46)*
False-positive biopsy recommendation rate (%)	2.75 (2.12, 3.57)	3.93 (2.39, 6.45)	1.18 (-0.45, 2.81)	0.77 (0.65, 0.91)	0.90 (0.72, 1.13)	0.13 (-0.06, 0.32)
Cancer detection rate (per 1,000 exams)	11.37 (9.23, 14.00)	12.65 (8.76, 18.26)	1.28 (-3.94, 6.50)	*5.23 (4.62, 5.91)*	*6.19 (5.44, 7.05)*	*0.97 (0.24, 1.70)*
Invasive cancer detection rate (per 1,000 exams)	8.71 (6.92, 10.98)	10.20 (7.51, 13.85)	1.49 (-2.10, 5.07)	**4.01 (3.57, 4.51)**	**4.84 (4.31, 5.43)**	**0.82 (0.35, 1.29)**
DCIS detection rate (per 1,000 exams)	2.65 (1.79, 3.92)	2.45 (0.31, 19.20)	-0.21 (-5.33, 4.91)	1.22 (0.99, 1.49)	1.36 (1.03, 1.80)	0.14 (-0.27, 0.55)
Cancer rate (per 1,000 exams)	11.80 (9.54, 14.59)	12.65 (8.76, 18.26)	0.85 (-4.46, 6.15)	*6.10 (5.42, 6.86)*	*7.07 (6.28, 7.96)*	*0.97 (0.17, 1.76)*
Interval cancer rate (per 1,000 exams)	--	--	--	0.87 (0.72, 1.06)	0.87 (0.64, 1.18)	0.00 (-0.29, 0.30)
Invasive interval cancer rate (per 1,000 exams)	--	--	--	0.77 (0.63, 0.94)	0.61 (0.45, 0.82)	-0.16 (-0.39, 0.06)
Total advanced cancer rate (per 1,000 exams)	2.27 (1.44, 3.58)	1.83 (0.48, 6.92)	-0.44 (-2.99, 2.12)	0.53 (0.43, 0.65)	0.40 (0.23, 0.69)	-0.13 (-0.39, 0.13)
Screen-detected advanced cancer rate (per 1,000 exams)	2.13 (1.34, 3.38)	1.83 (0.48, 6.92)	-0.29 (-2.83, 2.24)	0.37 (0.29, 0.48)	0.28 (0.15, 0.53)	-0.09 (-0.31, 0.13)
Interval advanced cancer rate (per 1,000 exams)	--	--	--	0.16 (0.11, 0.21)	0.12 (0.07, 0.20)	-0.04 (-0.12, 0.04)
PPV1 (recall) (%)	4.55 (3.57, 5.79)	6.10 (4.15, 8.96)	1.55 (-1.15, 4.25)	**6.33 (5.63, 7.11)**	**8.87 (7.63, 10.32)**	**2.54 (1.39, 3.69)**
PPV2 (biopsy recommended) (%)	23.09 (18.03, 29.57)	21.89 (14.12, 33.94)	-1.20 (-11.60, 9.21)	32.29 (29.06, 35.88)	33.61 (27.86, 40.54)	1.31 (-4.19, 6.82)
PPV3 (biopsy performed) (%)	26.96 (20.98, 34.65)	21.58 (11.93, 39.06)	-5.38 (-18.83, 8.07)	37.14 (33.66, 40.99)	38.43 (33.17, 44.51)	1.28 (-4.13, 6.69)
Sensitivity (%)	--	--	--	85.74 (83.57, 87.97)	87.29 (83.22, 91.56)	1.55 (-2.83, 5.93)
Specificity (%)	75.57 (72.58, 78.68)	80.28 (75.42, 85.46)	4.71 (-0.17, 9.60)	**92.07 (91.30, 92.84)**	**93.42 (92.67, 94.17)**	**1.35 (0.56, 2.14)**

Bold: absolute risk difference is significant with false discovery rate threshold of 0.05 after adjusting for multiple comparison (using Benjamini–Hochberg procedure). *Italic*: estimates with corresponding 95% CI of the absolute risk difference not covering 0, but not significant with false discovery rate threshold of 0.05 after adjusting for multiple comparison (using Benjamini–Hochberg procedure).
Models are adjusted for age group, category of FHBC, ethnicity and race, history of benign breast biopsy, breast density, screening interval, screening round, and BCSC participating registry.
Data in parenthesis are 95% confidence intervals. DBT: digital breast tomosynthesis, DM: digital mammography, DCIS: ductal carcinoma in situ, PPV: positive predictive values.

© 2025 Li T et al. *JAMA Oncology*.

eTable 13: Sensitivity analysis of characteristics of screen-detected and interval cancers by modality when restricting to women aged 50-74 according to age at the most recent screening prior to diagnosis

Cancer characteristic	Screen-detected cancers Number (%)		Interval cancers Number (%)	
**ALL CANCERS (N=2131)**	**DM (N=1341)**	**DBT (N=503)**	**DM (N=215)**	**DBT (N=72)**
**Histology type**				
Missing	0	0	0	1
DCIS	318 (23.7%)	109 (21.7%)	26 (12.1%)	10 (14.1%)
Invasive	1023 (76.3%)	394 (78.3%)	189 (87.9%)	61 (85.9%)
**AJCC anatomic stage**				
Missing	28	20	4	3
0	318 (24.2%)	109 (22.6%)	26 (12.3%)	10 (14.5%)
I	696 (53.0%)	288 (59.6%)	97 (46.0%)	35 (50.7%)
IIA	192 (14.6%)	58 (12.0%)	43 (20.4%)	11 (15.9%)
IIB	58 (4.4%)	17 (3.5%)	25 (11.8%)	9 (13.0%)
III-IV	49 (3.7%)	11 (2.3%)	20 (9.5%)	4 (5.8%)
**AJCC pathologic prognostic stage**				
Missing	102	58	44	10
0	318 (25.7%)	109 (24.5%)	26 (15.2%)	10 (16.1%)
I	837 (67.6%)	315 (70.8%)	117 (68.4%)	42 (67.7%)
IIA	43 (3.5%)	15 (3.4%)	9 (5.3%)	6 (9.7%)
IIB	11 (0.9%)	3 (0.7%)	6 (3.5%)	1 (1.6%)
III-IV	30 (2.4%)	3 (0.7%)	13 (7.6%)	3 (4.8%)
**INVASIVE CANCERS (N=1667)**	**DM (N=1023)**	**DBT (N=394)**	**DM (N=189)**	**DBT (N=61)**
**Histologic grade**				
Missing	27	5	10	1
1	340 (34.1%)	141 (36.2%)	46 (25.7%)	20 (33.3%)
2	423 (42.5%)	162 (41.6%)	74 (41.3%)	20 (33.3%)
3	233 (23.4%)	86 (22.1%)	59 (33.0%)	20 (33.3%)
**Invasive cancer size**				
Missing	29	11	6	2
<6mm	147 (14.8%)	59 (15.4%)	16 (8.7%)	7 (11.9%)
6-10mm	256 (25.8%)	117 (30.5%)	22 (12.0%)	11 (18.6%)
11-15mm	262 (26.4%)	107 (27.9%)	50 (27.3%)	11 (18.6%)
16-20mm	118 (11.9%)	38 (9.9%)	22 (12.0%)	9 (15.3%)
>20mm	211 (21.2%)	62 (16.2%)	73 (39.9%)	21 (35.6%)
**Axillary lymph node status**				
Missing	23	9	3	2
Negative for metastases	799 (79.9%)	326 (84.7%)	130 (69.9%)	44 (74.6%)
Positive for metastases	201 (20.1%)	59 (15.3%)	56 (30.1%)	15 (25.4%)

© 2025 Li T et al. *JAMA Oncology*.

**Estrogen and progesterone receptor status**				
Missing	13	12	2	3
ER+ or PR+	901 (89.2%)	351 (91.9%)	161 (86.1%)	42 (72.4%)
ER- and PR-	109 (10.8%)	31 (8.1%)	26 (13.9%)	16 (27.6%)
**HER2 status**				
Missing	74	31	17	4
Positive	101 (10.6%)	29 (8.0%)	19 (11.0%)	12 (21.1%)
Negative	848 (89.4%)	334 (92.0%)	153 (89.0%)	45 (78.9%)

Note: For parameters with missing data, percentages were calculated among records with known values.

© 2025 Li T et al. *JAMA Oncology*.
