# Supplementary material for: Tomosynthesis vs Digital Mammography Screening in Women with a Family History of Breast Cancer
Source: JAMA Oncol. 2025 May 22;11(7):742–52. doi: 10.1001/jamaoncol.2025.1209 (PMC12100508; doi:10.1001/jamaoncol.2025.1209)
Supplement: Supplement 2. — Data Sharing Statement [file jamaoncol-e251209-s002.pdf]

Data Sharing Statement

Li. Tomosynthesis vs Digital Mammography Screening in Women with a Family History of Breast Cancer. *JAMA Oncol*. Published May 22, 2025. doi:10.1001/jamaoncol.2025.1209

Data

Data available: No

Additional Information

Explanation for why data not available: Study protocol and statistical code available on request; please contact kpwa.scc@kp.org with specific queries. Data will be available after study aims of funded grants are addressed and with appropriate regulatory approvals.
